# Transcriptomic features of primary prostate cancer and their prognostic relevance to castration-resistant prostate cancer

**DOI:** 10.18632/oncotarget.22296

**Published:** 2017-11-06

**Authors:** Seok Joong Yun, Seon-Kyu Kim, Jayoung Kim, Eun-Jong Cha, Jang-Seong Kim, Sun-Jin Kim, Yun-Sok Ha, Ye-Hwan Kim, Pildu Jeong, Ho Won Kang, Jeong-Hwan Kim, Jong-Lyul Park, Young-Ki Choi, Sung-Kwon Moon, Yung-Hyun Choi, Seon-Young Kim, Wun-Jae Kim

**Affiliations:** ^1^ Department of Urology, Chungbuk National University College of Medicine, Cheongju, Korea; ^2^ Department of Urology, Chungbuk National University Hospital, Cheongju, Korea; ^3^ Personalized Genomic Medicine Research Center, Korea Research Institute of Bioscience and Biotechnology, Daejeon, Korea; ^4^ Department of Surgery, Harvard Medical School, Boston, MA, USA; ^5^ Division of Cancer Biology and Therapeutics, Departments of Surgery and Biomedical Sciences, Samuel Oschin Comprehensive Cancer Institute, Cedars-Sinai Medical Center, Los Angeles, CA, USA; ^6^ Department of Medicine, University of California Los Angeles, Los Angeles, CA, USA; ^7^ Department of Biomedical Engineering, Chungbuk National University College of Medicine, Cheongju, Korea; ^8^ Biotherapeutics Translational Research Center, Korea Research Institute of Bioscience and Biotechnology, Daejeon, Korea; ^9^ R&D Center, Hanmi Pharm. Co. Ltd., Hwaseong-si, Korea; ^10^ Department of Urology, School of Medicine, Kyungpook National University, Daegu, Korea; ^11^ Department of Microbiology, College of Medicine and Medical Research Institute, Chungbuk National University, Cheongju, Korea; ^12^ School of Food Science and Technology, Chung-Ang University, Anseong, Korea; ^13^ Department of Biochemistry, Dongeui University College of Oriental Medicine, Busan, Korea; ^14^ Department of Functional Genomics, University of Science and Technology, Daejeon, Korea

**Keywords:** CRPC, disease progression, markers, prognosis, gene expression

## Abstract

Although various mechanisms of castration-resistant prostate cancer (CRPC) have been discovered, reliable biomarkers for monitoring CRPC progression are lacking. We sought to identify molecules that predict the progression of advanced prostate cancer (AdvPC) into CRPC. The study used primary-site samples (*N*=45 for next-generation sequencing (NGS); *N*=243 for real-time polymerase chain reaction) from patients with prostate cancer (PC). Five public databases containing microarray data of AdvPC and CRPC samples were analyzed. The NGS data showed that each progression step in PC associated with distinct gene expression profiles. *Androgen receptor* (*AR*) associated with tumorigenesis, advanced progression, and progression into CRPC. Analysis of the paired and unpaired AdvPC and CRPC samples in the NGS cohort showed that 15 genes associated with progression into CRPC. This was validated by cohort-1 and public database analyses. Analysis of the third cohort with AdvPC showed that higher *serine peptidase inhibitor, Kazal type 1* (*SPINK1*) and lower *Sp8 transcription factor* (*SP8*) expression associated with progression into CRPC (log-rank test, both *P*<0.05). Multivariate regression analysis showed that higher *SPINK1* (Hazard Ratio (HR)=4.506, 95% confidence intervals (CI)=1.175–17.29, *P*=0.028) and lower *SP8* (HR=0.199, 95% CI=0.063–0.632, *P*=0.006) expression independently predicted progression into CRPC. Gene network analysis showed that CRPC progression may be mediated through the *AR-SPINK1* pathway by a *HNF1A*-based gene network. Taken together, our results suggest that*SPINK1* and *SP8* may be useful for classifying patients with AdvPC who have a higher risk of progressing to CRPC.

## INTRODUCTION

Prostate cancer (PC) is currently the most common malignancy in males [1]. Unfortunately, about 20% of all PC patients will present with or develop disease progression and metastasis [2, 3]. For patients with such advanced PC (AdvPC), the therapeutic options include prostatectomy, radiation therapy, and/or androgen-deprivation therapy (ADT) [4]. However, despite these therapies, the prognosis of AdvPC remains poor: for example, the 5-year survival rate of patients with AdvPC who are treated with ADT is 23–26% [3]. This is because ADT can only induce short 2–3 year remissions since most PCs eventually develop into castration-resistant prostate cancer (CRPC) [3]. CRPC associates with a particularly poor prognosis. The mechanism by which PC cells become castration-resistant remains unclear. After PC cells acquire castration resistance, the treatment options are limited to second-line ADT (abiraterone or enzalutamide), new generation cytotoxic chemotherapy, or secondary symptomatic relief of bone metastases [5]. Thus, the management of AdvPC and CRPC remains difficult. There is a great need for robust methods that can identify AdvPC patients who have a higher risk of progressing to CRPC.

Recent advances in high-throughput next-generation sequencing (NGS) technologies have greatly improved our understanding of the genomics and epigenomics of PC [6–9]. In addition, integrative genomic approaches have revealed various novel molecular characteristics of CRPC [7, 9]. However, these studies have only identified a handful of reliable and practical criteria that can adequately predict progression of AdvPC. Moreover, the vast majority of these studies were conducted on metastasized CRPC discovered at distant sites. Since these sites are not the primary tumor site and metastatic sites have completely different microenvironments from the primary site [10], these studies do not reflect the precise molecular or clinical characteristics of progression of AdvPC into CRPC.

Here, we used NGS to explore the transcriptomes of primary-site samples from patients with PC at various stages of progression, namely, benign prostate hyperplasia (BPH), localized PC, AdvPC, and CRPC. The findings were validated by analyses of public databases containing microarray data of AdvPC and CRPC samples, and by subjecting primary-site samples from an independent cohort of patients with various PC stages to reverse transcriptase-polymerase chain reaction (RT-PCR) analysis. A third independent cohort of patients with AdvPC who underwent surgery and then prolonged follow-up was used to assess the ability of selected genes to predict progression of AdvPC into CRPC.

## RESULTS

### PC progression steps associate with distinct gene-expression profiles

All patients’ characteristics were described in Table 1. The transcriptomes of 45 prostate samples from the NGS cohort were obtained by RNA-Seq. Unsupervised hierarchical clustering analysis yielded three main sample clusters that consisted of all BPH (normal) samples, all localized PC (T2 or T3 N0 M0) samples, and all AdvPC (T4 or any N1/M1) plus CRPC samples (Supplementary Figure 1). The AdvPC and CRPC samples were not clearly distinguishable from one another. Thus, as AdvPC progresses into CRPC, only a few genes change their expression significantly. By contrast, larger transcriptome changes are observed when BPH compares to localized PC and when localized PC compares to AdvPC.

**Table 1:** Clinical characteristics of the patients and controls in the NGS cohort, the validation cohort, and the prognostic cohort

Characteristic	NGS cohort				Validation cohort				Prognostic cohort
	BPH controls	Localized PC	AdvPC	CRPC	BPH controls	Locally advanced or AdvPC	Hormone-suppressed PC	CRPC	Locally advanced or AdvPC
No.	8	16	9^*^	12^*^	58	62	14	15	94
Age, years, mean (range)	65.1 (54–70)	68.1 (54–75)	74.9 (69–82)	73.7 (59–82)	71.5 (54–90)	71.4 (48–85)	72.2 (52–78)	69.6 (53–85)	69.9 (52–86)
PSA at operation, ng/ml, mean±SD	1.8±0.4	15.1±7.9	332.8±276.0	62.3±56.0	1.7±1.3	387.9±1239.3	9.8±15.7	193.6±387.3	277.8±959.5
Operation, *n* (%)									
TUR-P	8	0	1	12	58	44 (71.0)	14 (100)	15 (100)	59 (62.8)
Radical prostatectomy	0	16	8	0		18 (29.0)	0 (0.0)	0 (0.0)	35 (37.2)
Gleason score, *n* (%)									
6 or less		2	0	0		5 (8.1)	0 (0.0)	0 (0.0)	0 (0.0)
7		11	3	2		20 (32.3)	2 (16.3)	3 (20.0)	42 (44.7)
8		0	0	2		14 (22.6)	3 (21.4)	1 (6.7)	17 (18.1)
9		3	6	7		21 (33.9)	8 (57.1)	8 (53.3)	32 (34.0)
10		0	0	1		2 (3.2)	1 (7.1)	3 (20.0)	3 (3.2)
TNM stage, *n* (%)									
T2 or T3, N0, M0		16	0	0		17 (27.4)	1 (7.1)	0 (0.0)	47 (50.0)
T4 or metastatic		0	9	12		45 (72.6)	13 (92.9)	15 (100)	47 (50.0)

^*^In the NGS cohort, four patients had biopsies of both their AdvPC and the CRPC that arose from it later.

Abbreviations: BPH, benign prostate hyperplasia; NGS, next-generation sequencing; PC, prostate cancer; AdvPC, advanced PC; PSA, prostate-specific antigen; SD, standard deviation; TUR-P, transurethral resection of the prostate.

**Figure 1:**
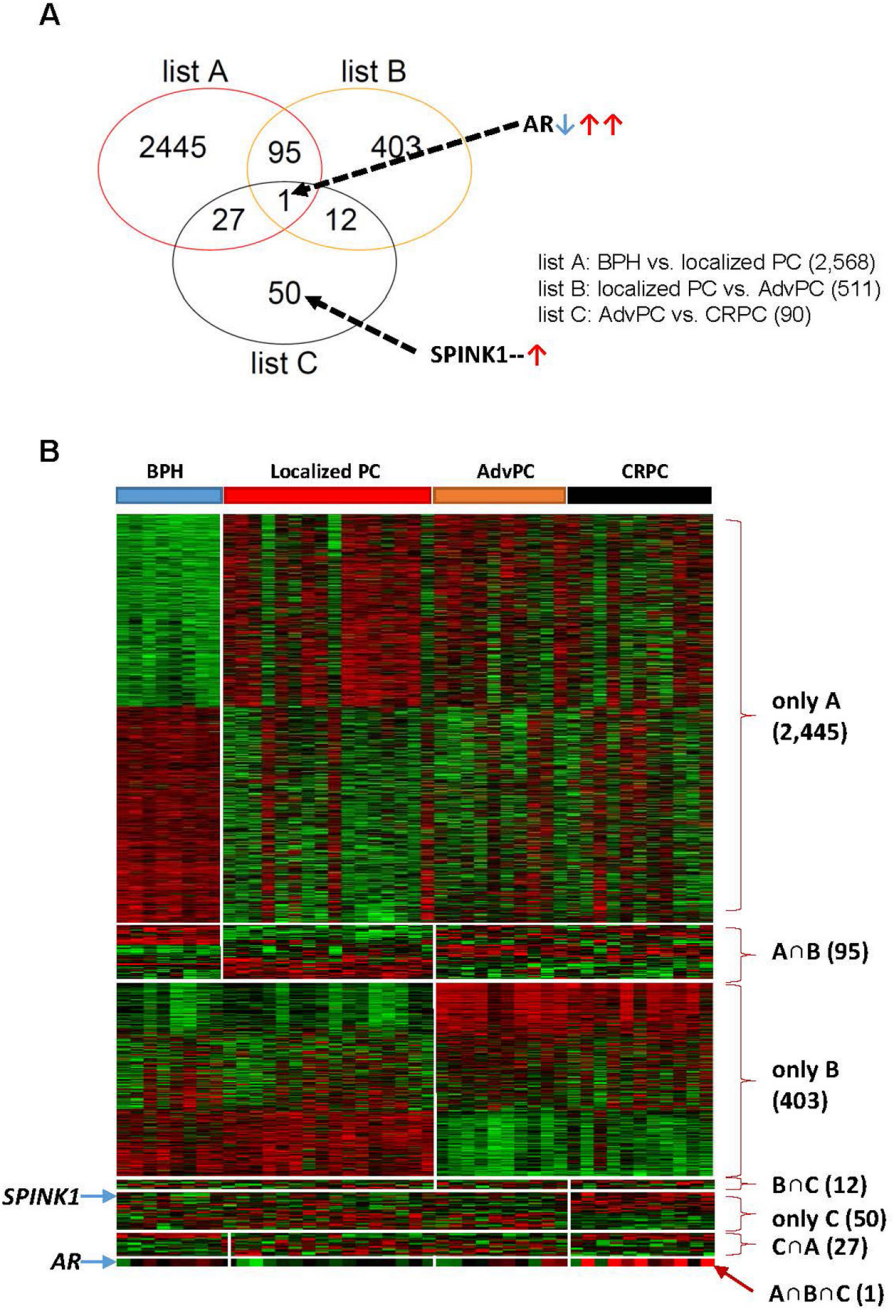
Differentially expressed genes in the prostate tissue samples from the next-generation sequencing (NGS) cohort The NGS cohort consisted of patients with benign prostate hyperplasia (BPH), localized prostate cancer (PC), advanced PC (AdvPC), or castration-resistant PC (CRPC). **(A)** Venn diagram of the genes that were differentially expressed in the BPH, localized PC, AdvPC, and CRPC patients. The genes were selected by using the GLM likelihood ratio test with EdgeR software. Genes whose differential expression was ≥2-fold and significant (*P*>0.001) were selected. Genes in the red circle (list A) indicate those that are differentially expressed between BPH and localized PC. Genes in the orange circle (list B) indicate those that are differentially expressed between localized PC and AdvPC. Genes in the black circle (list C) indicate those that are differentially expressed between AdvPC and CRPC. **(B)** Expression patterns of selected genes in the Venn diagram. The data are presented in matrix format where rows indicate individual genes and columns show the indicated tissue. Red and green colors indicate high and low expression, respectively.

The differentially expressed genes in the BPH, localized PC, AdvPC, and CRPC samples were determined by using the GLM likelihood ratio test. This yielded three gene lists that showed the genes whose expression changed when BPH compared to localized PC (list A), localized PC compared to AdvPC (list B), and AdvPC compared to CRPC (list C) (*P*<0.001, Supplementary Figure 2). Similar to the hierarchical cluster analysis, the transcriptomes of AdvPC and CRPC only differed by 90 genes. Greater differences between transcriptomes were observed in the other comparisons. A Venn diagram was then used to compare the three gene lists (Figure 1A). Several different patterns were observed: A only (BPH *vs*. localized PC: 2,445 genes), B only (localized PC *vs*. AdvPC: 403 genes), C only (AdvPC *vs*. CRPC: 50 genes), A∩B (95 genes), B∩C (12 genes), C∩A (27 genes), and A∩B∩C (one gene) (Figure 1B). Thus, the genes in the A and B only categories had expression patterns that associated with tumorigenesis and advanced progression, respectively; the genes in the C only category had expression patterns that associated with progression into CRPC; the genes in the A∩B category were common to tumorigenesis and advanced progression; the genes in the B∩C category were common to advanced progression and CRPC development; and the genes in the C∩A category were common to tumorigenesis and CRPC development. The only gene that associated with tumorigenesis, advanced progression, and CRPC development was *AR* (Figure 1): when tumorigenesis transformed BPH into localized PC, *AR* was significantly down-regulated. By contrast, it was up-regulated when localized PC progressed to AdvPC and further upregulated when AdvPC progressed into CRPC (Supplementary Figure 2). Thus, *AR* could be a crucial mediator of PC progression.

### Progression of AdvPC into CRPC associates with expression changes in 12 genes

The NGS cohort samples included four pairs of AdvPC and CRPC samples from the same patient. These eight paired samples were assessed for genes that changed their expression when AdvPC progressed into CRPC: 309 genes were significantly differentially expressed. A similar analysis of the 13 unpaired AdvPC and CRPC samples in the NGS cohort showed that 182 genes were significantly differentially expressed (*P*<0.001 by the GLM likelihood ratio test). Comparison of these two gene lists showed 15 genes in common (Supplementary Figure 3). Of these 15, two (*AR* and *SPINK1*) were up-regulated in CRPC compared to in AdvPC and ten (*ALOX15B*, *ANPEP*, *CBLN2*, *CEACAM20*, *CEACAM22P*, *SERPINB11*, *SNCA*, *SP8*, *TRPM8*, and *WNT11*) were down-regulated in CRPC compared to in AdvPC (Supplementary Table 3). The remaining genes (*KLK1*, *PGC*, and *PPFIA2*) showed different expression changes in the paired and unpaired samples and were not considered further.

### Confirmation of the 12 CRPC development-associated genes

Public gene-expression datasets were used to verify the expression patterns of the 12 genes whose expression changed significantly as AdvPC progressed into CRPC. The available probes that matched with the 12 genes were used only. In the GSE28403 dataset, comparison of the four AdvPC and nine CRPC samples in terms of gene expression showed that apart from a few exceptions, most gene expression changes were consistent with those seen in our cohort. However, the gene expression differences seen in GSE28403 only achieved statistical significance a few times (one was *AR*; Supplementary Figure 4). This may be due to the small sample size of GSE28403 (*N*=13) and the fact that all AdvPC and two CRPC samples were from metastatic sites.

A second verification was performed with the GSE32269 dataset, which included 21 primary-site localized PC and 29 metastatic CRPC samples. Again, most expression changes were consistent with those seen in our cohort. Several changes achieved statistical significance (one was *AR*; Supplementary Figure 5).

In a third dataset, GSE35988, which included 59 primary-site localized PC and 35 metastatic CRPC samples, all but one gene exhibited expression changes that were consistent with those seen in our cohort. There were also more statistically significant changes in this cohort than in the other cohorts (one was *AR*; Supplementary Figure 6).

**Figure 2:**
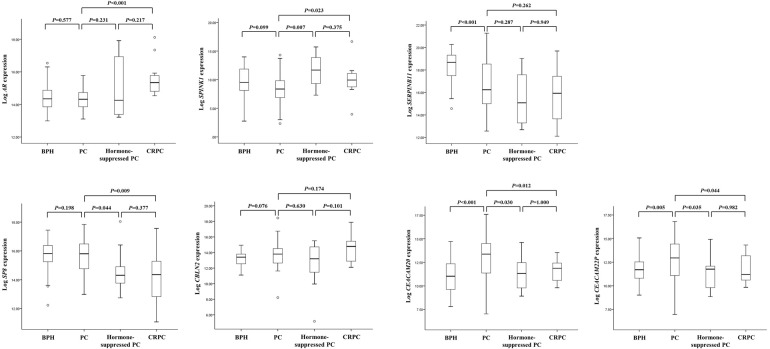
The seven genes that were differentially expressed between primary-site advanced prostate cancer (PC) and castration-resistant PC (CRPC) samples in the NGS cohort were validated by analyzing the validation cohort by using real-time PCR The cohort consisted of patients with benign prostate hyperplasia (BPH), locally advanced or advanced PC (AdvPC), hormone-suppressed PC, or CRPC. The patterns of *AR*, *SPINK1*, *SP8*, *CEACAM20*, and *CEACAM22P* expression were consistent with those observed in the NGS cohort.

To further confirm that the expression of these genes changed as AdvPC progressed into CRPC, we subjected primary-site PC samples from a second independent cohort to RT-PCR analysis. These samples consisted of BPH, locally advanced or AdvPC, hormone-suppressed PC, and CRPC samples. We only assessed 7 of the 12 genes because previous studies have already shown that *ALOX15B*, *ANPEP*, *SNCA*, *TRPM8*, and *WNT11* associate strongly with AdvPC [11–16]. Of these seven genes, two (*AR* and *SPINK1*) were up-regulated by progression into CRPC and five (*SERPINB11*, *SP8*, *CBLN2*, *CEACAM20*, and *CEACAM22P*) were down-regulated. BPH only differed significantly from the PC samples in terms of *SERPINB11*, *CEACAM20*, and *CEACAM22P* expression (Figure 2). By contrast, the three PC stages differed significantly from each other in terms of *AR*, *SPINK1*, *SP8*, *CEACAM20*, and *CEACAM22P* (but not *SERPINB11* and *CBLN2*) expression (Figure 2). Thus, these data corresponded largely with the RNA-Seq data from the NGS cohort.

### Two genes can predict the risk of progression into CRPC

**Figure 3:**
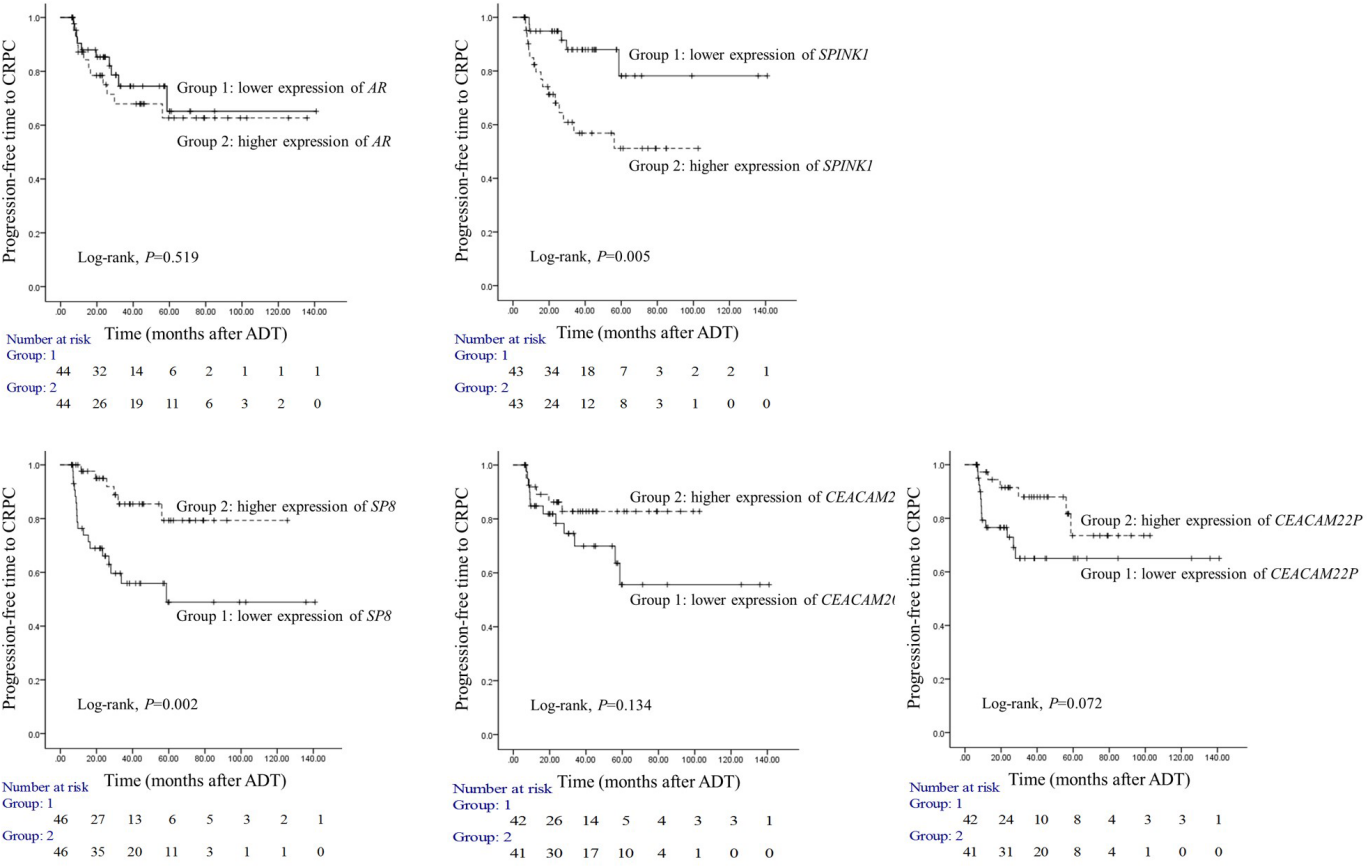
Prognostic usefulness of five genes that associated with progression from locally advanced or advanced prostate cancer (PC) to castration-resistant PC (CRPC) The prognostic cohort consisted of 94 patients with locally advanced or advanced PC (AdvPC) who underwent surgery followed by androgen-deprivation therapy (ADT) and were followed up on average for 32.7 months. The patients were divided into two groups depending on whether they had higher or lower *AR*, *SPINK1*, *SP8*, *CEACAM20*, or *CEACAM22P* expression. The time to CRPC in the two subgroups for each gene was analyzed. Higher *SPINK1* and lower *SP8* expression associated with progression to CRPC.

To determine the prognostic usefulness of the five genes that associated significantly with progression to CRPC in our validation cohort analysis, we employed our third independent cohort. All patients in this cohort underwent surgery for AdvPC and were placed on ADT and followed on average for 32.7 months. Of the 94 patients, 24 developed CRPC during follow-up. We analyzed the 94 primary-site AdvPC samples that were taken at surgery for the expression of the five candidate genes and then divided the patients into two groups on the basis on the expression of each gene by median values. The progression-free survival duration of the two groups (*i.e.*, duration between starting ADT and CRPC) was then analyzed (Figure 3). Only *SPINK1* and *SP8* were significantly predictive of time to CRPC. Patients with higher *SPINK1* expression progressed to CRPC significantly more frequently than patients with lower *SPINK1* expression (log-rank test, *P*=0.005). Similarly, patients with lower *SP8* expression progressed to CRPC significantly more frequently than patients with higher *SP8* expression (log-rank test, *P*=0.002).

To verify the prognostic usefulness of *SPINK1* and *SP8*, we subjected the third cohort data to multivariate Cox regression analysis to determine the independent association between *SPINK1* and *SP8* with known clinicopathological risk factors of PC. Even after applying a variable selection procedure, type of operation, lymph node or distant metastasis, higher *SPINK1* expression (HR=4.506, 95% CI=1.175–17.29, *P*=0.028), and lower *SP8* expression (HR=0.199, 95% CI=0.063–0.632, *P*=0.006) were independent risk factors for CRPC progression (Table 2).

**Table 2:** Multivariate Cox regression modeling to identify risk factors for the progression of AdvPC into CRPC

Variable	Univariate analysis		Multivariate analysis	
	HR (95% CI)	*P*	HR (95% CI)	*P*
Type of operation (RP *vs*. TUR–P)	8.02 (3.277–19.631)	<0.001	3.572 (1.074–11.882)	0.038
PSA (<20 *vs*. ≥20 ng/mL)	4.718 (1.407–15.82)	0.012		
Lymph node or distant metastasis (no *vs*. yes)	8.322 (2.831–24.462)	<0.001	7.076 (1.435–34.886)	0.016
Gleason score (7 *vs*. 8–10)	3.892 (1.451–10.439)	0.007		
*SPINK1* expression (lower *vs*. higher)^*^	3.84 (1.404–10.503)	0.009	4.506 (1.175–17.29)	0.028
*SP8* expression (lower *vs*. higher)^*^	0.256 (0.101–0.646)	0.004	0.199 (0.063–0.632)	0.006

Abbreviations: AdvPC, advanced prostate cancer; CRPC, castration-resistant prostate cancer; CI, confidence interval; HR, hazard ratio; PSA, prostate-specific antigen; RP, radical prostatectomy; TUR–P, transurethral resection of the prostate.

^*^Expressions of gene were divided by medial value.

### Biological insights into the gene profile of progression into CRPC

**Figure 4:**
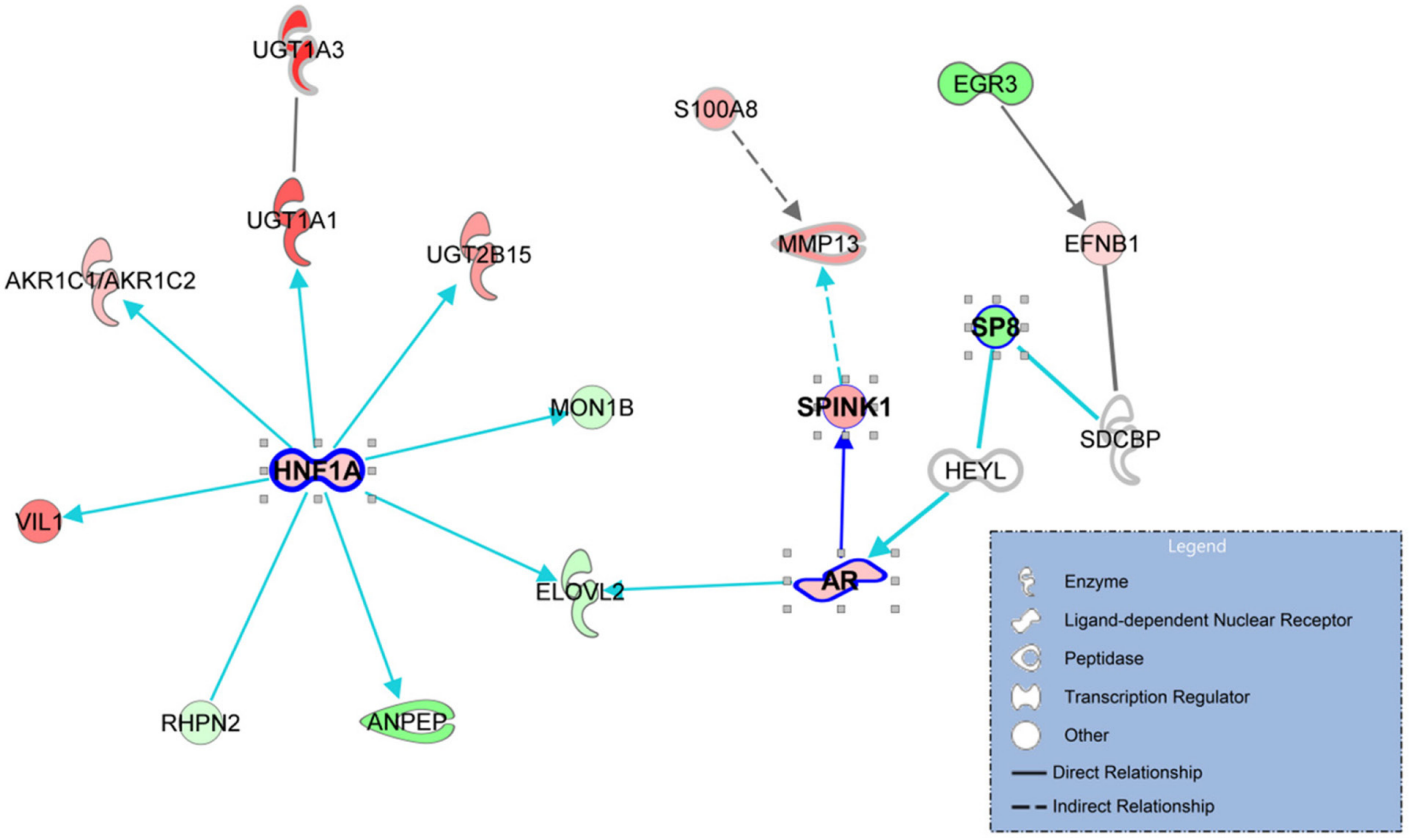
The gene networks that are enriched with the genes that associated with progression to castration-resistant prostate cancer (CRPC) The 90 genes that were differentially expressed between advanced prostate cancer (PC) and CRPC were used to explore known gene networks. The genes that were up- and down-regulated in CRPC relative to advanced PC (AdvPC) are indicated by red and green, respectively. The color intensity indicates the degree of up- or down-expression. Each line and arrow indicates functional and physical interactions between the genes and the direction of regulation that was reported in the literature.

To identify signaling pathways that promote progression to CRPC, a gene-to-gene network analysis of the 90 genes that were differentially expressed between AdvPC and CRPC sample groups (Figure 1) was performed by using the IPA tool. Analysis of literature-based gene networks revealed functional connectivity between *AR* and *SPINK1*, which indirectly regulated by *SP8*, and a putative gene network hub mediated by *HNF1A* (Figure 4). Analysis of publicly available datasets from five independent cohorts of patients with AdvPC or CRPC (including the three described above) confirmed that expression changes of *AR*, *SPINK1*, *HNF1A*, and several other network members were well agreed with those in the NGS cohort. (Supplementary Figures 8–12). Details are available in the Supplementary Materials.

## DISCUSSION

PC is a heterogeneous disease with diverse progression steps that involves the activities of various molecules. Identification of the genes that associate specifically with the progression of AdvPC into CRPC will help identify the patients at risk of such progression and will aid the development of therapeutic strategies that block this progression step. Such therapies could greatly improve the overall quality of life of PC patients and prolong their survival. With this in mind, we subjected 45 primary-site samples from patients with BPH, localized PC, AdvPC, or CRPC to transcriptome analysis to identify the genes that associate with each PC progression step. Transcriptome profiling showed that 90 genes significantly associated with progression from AdvPC to CPRC. In particular, one gene, *AR*, was down-regulated when BPH compared to localized PC, up-regulated when localized PC compared to AdvPC, and further up-regulated when AdvPC compared to CRPC. Thus, *AR* may participate in all PC progression steps. Bioinformatic- and RT-PCR-based experiments with four independent patient cohorts confirmed the significant role of five additional genes in progression to CRPC. An analysis of an independent cohort of patients with AdvPC who underwent surgery, ADT, and extensive follow-up then showed that the expression patterns of two of these five genes, *SPINK1* and *SP8*, in the primary site predicted progression into CRPC: specifically, higher *SPINK1* and lower *SP8* expression associated with shorter times to CRPC development. Multivariate regression analysis confirmed that these genes are independent predictors of the risk of progression to CRPC, even after considering clinicopathological indicators. Gene network analysis also revealed signaling pathways that may be responsible for CRPC development.

*SPINK1* encodes a trypsin inhibitor that is secreted by pancreatic acinar cells. Mutations in *SPINK1* associate with hereditary pancreatitis and tropical calcific pancreatitis [17]. *SPINK1* also participates in several important cellular activities, including abnormal morphology and proliferation. Moreover, it is up-regulated in many cancers, including PC [18–23]. There are several other biological links between *SPINK1* and PC. First, Tomlins *et al.* showed that *SPINK1* is highly up-regulated in a molecular subtype of ETS-rearrangement-negative PC and that *SPINK1* was an independent predictor of biochemical recurrence of these PCs [23]. However, Flavin *et al.* showed that *SPINK1*-encoded protein may not predict biochemical recurrence or lethal PC after radical prostatectomy [22]. This disparity may reflect the different composition of the patient cohorts in these two studies [23]. Second, *SPINK1* is directly regulated by *AR* in prostate tissue and has been reported to be a potential therapeutic target in CRPC [3, 24]. However, we are the first to show that *SPINK1* is of prognostic relevance in CRPC.

We also showed for the first time that *SP8* may be an independent indicator of CRPC progression as well. *SP8* is a SP family transcription factor that plays an essential role in proper limb development. There is also an association between *SP8* and the development of other urological diseases such as hypospadias [25]. However, there are very few reports on its role in the cell or its association with cancer.

We used public datasets to validate our finding that progression of PC into CRPC associated with expression changes in several genes. While the public datasets did generally confirm our findings, there were some discrepancies. First, while *SPINK1* did tend to be up-regulated in CRPC in the public datasets relative to advanced or localized PC, this change never achieved statistical significance (Supplementary Figures 4–6 and 8–12). By contrast, it was strongly up-regulated in the CRPCs in validation cohort-1 (Figure 2). Second, although *SP8* was down-regulated in CRPC in the NGS cohort relative to AdvPC, it was up-regulated in the CRPCs in the GSE28403 dataset relative to AdvPC (Supplementary Figure 4). This may be due to the small sample size of that public dataset and the fact that all AdvPCs and two of the nine CRPCs were from metastatic sites. Indeed, our analysis of the validation cohort confirmed that *SP8* is down-regulated in CRPCs (Figure 2). These results underscore the importance of having a sufficient sample size and maintaining a consistent sampling method when obtaining tissue samples for genomic comparisons.

Our gene-to-gene network analysis of the 90 genes that were differentially expressed between AdvPC and CRPC revealed a putative *HNF1A-*mediated gene network and functional connectivity between *AR* and *SPINK1* (Figure 4). The genes that are regulated by *HNF1A* include UDP glucuronosyltransferase (UGT) family members, namely, *UGT1A1*, *UGT1A3*, and *UGT2B15*. Like *HNF1A*, these proteins participate in cancer, cell proliferation, and tumor morphology. Metabolomic profiling of PC has also shown that CRPCs differ significantly from androgen-dependent PC in terms of UGT activity-associated metabolites [26]. Thus, UGT activity may be important for modulating the activity of androgens [27]. The pathway that is regulated by UGT activity may also be a potential source of targets for drug discovery in CRPC [3]. Notably, when we explored the role of hormone interactions in our gene-to-gene networks, we found that many hormones mediated the signaling of *AR* and the downstream effectors of the *HNF1A* network, including the UGT family members (Supplementary Figure 13). This suggests that the *AR*- and *HNF1A*-mediated gene networks participate in the development of CRPC. However, the precise role that *HNF1A* and these hormone interactions play in CRPC development remains to be determined experimentally, because these results were derived from only computational analysis.

There were several limitations in our study. First, our patient cohorts only contained 27 CRPC samples. This is insufficient for rigorously determining the role of genes in progression to CRPC. Second, we analyzed the transcriptomics datasets without considering other biological events such as mutations, copy number alteration, or epigenetic changes. This may have hampered our mechanistic insights into CRPC development. Third, our analyses only provided indirect evidence for the role of *HNF1A* and its network member genes in CRPC progression; these activities must be validated by biological assays. Forth, we used small part of whole cancer tissues, and some of harvested tissues could not explain the all cancer clones because of tumor heterogeneity of PC. Lastly, BPH tissues could not represent the normal prostate tissue, because the most of cancer and BPH are arose from the different zone of the prostate.

Despite these limitations, however, this investigation has several key advantages over similar studies. First, AdvPC and CRPC sample pairs were obtained from four patients and all methods were handled by a single institute, including extraction of patient clinical information, RNA extraction, and data processing; this is likely to have limited heterogeneity between patient samples. Second, the gene-expression profiles that associated with three PC progression steps were determined. Third, to ensure that we obtained a gene profile that associated specifically with the development of CRPC, we only tested primary CRPCs that were obtained from homogenous biopsy sites; none of our PC samples were from distant tissue sites.

In conclusion, during our analysis of the transcriptomic changes that occur during various multiple PC progression steps, two genes, namely, *SPINK1* and *SP8*, were found to be reliable prognostic indicators of progression to CRPC; these markers are independent of the classical pathological prognostic variables.

## MATERIALS AND METHODS

### Patients and tissue samples

Our cohort study was based on 288 primary-site samples from three independent cohorts of PC patients. All samples were obtained from patients treated at the Chungbuk National University Hospital. All tumor samples were acquired after patient consent for tissue sample donation and examination was received. The patients who received radiation therapy were excluded for the study. The study protocol was approved by the Institutional Review Board for Human Genetic and Genomic Research (IRB approval number: 2006-01-001 and GR2010-12-010). PC was defined as CRPC if the prostate-specific antigen (PSA) levels had risen three times in a row despite the patient having low serum testosterone levels (<50 ng/mL).

The first cohort consisted of 45 prostate tissue samples (eight BPHs, 16 localized PCs, nine AdvPCs, and 12 CRPCs) from 41 patients (Table 1). Four patients provided both AdvPC and CRPC samples at different time points (Supplementary Table 1). All samples were subjected to NGS to explore their transcriptomes. It should be emphasized that all samples in this cohort were collected from the primary cancer site in the prostate: none were metastasized samples that were discovered at distant tissue sites.

The second cohort was a validation cohort. It consisted of 58 non-cancer BPHs, 62 locally advanced or AdvPCs, 14 hormone-suppressed PCs, and 15 CRPCs (Table 1). Hormone-suppressed PC was defined as PC when the patient was receiving ADT and there was no indication of progression into CRPC.

The third cohort was used to determine the prognostic value of selected genes. It consisted of 94 patients with locally advanced or advanced disease who received ADT after surgery (Table 1). Of these patients, 24 developed CRPC within 32.7 months of follow up (6.1–140 months).

### RNA-Seq experiments and data processing

RNA extraction and preparation for RNA-Seq was performed as described (Supplementary Materials).

### Public gene-expression datasets

To validate the NGS data, five gene-expression datasets (GSE28403, GSE32269, GSE35988, GSE37199, and GSE70768) that were based on microarray analysis of samples of PC at various progression stages were obtained from the National Center for Biotechnology (NCBI) Gene Expression Omnibus (GEO) [9, 28–31]. These cohorts are described in the Supplementary Materials.

### Real-time PCR

mRNA levels were measured by RT-PCR as described (Supplementary Materials).

### Statistical analysis

Statistical analyses were performed by using R (ver. 3.2.5.) and SPSS (ver. 23). To determine the mRNA-expression profiles of the prostate samples, a hierarchical clustering algorithm with centered correlation coefficient for similarity measure and complete linkage clustering was used. For clustering, the fragments per kilobase of transcript per million fragments mapped (FPKM) of each sample were used to estimate each gene expression. The FPKM data was normalized by the quantile method, log2-transformed, and median-centered across genes and samples.

To compare sample subgroups in the NGS cohort in terms of gene expression, an EdgeR package that utilizes a negative binomial model was used to detect differentially expressed genes from count data [32]. The gene count dispersion was estimated by using a Cox-Reid profile-adjusted likelihood method. After model fitting and estimation of dispersion, differentially expressed genes were selected by using a generalized linear model (GLM) likelihood ratio test. To verify gene expression differences between sample subgroups in the validation cohorts, two-sample *t*-tests were performed for each gene. Expression differences in genes were considered statistically significant if *P* was <0.001 and the fold difference in expression between two sample groups was ≥2. The Kaplan−Meier method was used to calculate the time to CRPC progression and the difference in survival between two groups was assessed by using log-rank statistics. The association between potential risk factors and prognosis was assessed by using multivariate Cox regression models. A backward-forward step procedure was applied to generate the multivariate model with the most informative variables [33]. The results were expressed as Hazard Ratio (HR) and 95% confidence intervals (CI).

Gene-set enrichment analysis was performed to identify the most significant gene sets that associated with PC progression. The significance of over-represented gene sets was estimated by Fisher’s exact test. To explore the relationships between the genes that associated with progression into CRPC, we searched for a connection between the genes by examining the previously reported literature and generated gene networks based on whether they had more connected genes than would be expected to occur by chance. Gene set enrichment and gene network analyses were performed by using the Ingenuity Pathway Analysis^™^ (IPA) tool.

## SUPPLEMENTARY MATERIALS FIGURES AND TABLES



## References

[1] Siegel RL, Miller KD, Jemal A (2017). Cancer statistics, 2017. CA Cancer J Clin.

[2] Prostate Cancer Trialists’ Collaborative Group (1995). Maximum androgen blockade in advanced prostate cancer: an overview of 22 randomised trials with 3283 deaths in 5710 patients. Lancet.

[3] Yap TA, Smith AD, Ferraldeschi R, Al-Lazikani B, Workman P, de Bono JS (2016). Drug discovery in advanced prostate cancer: translating biology into therapy. Nat Rev Drug Discov.

[4] Droz JP, Aapro M, Balducci L, Boyle H, Van den Broeck T, Cathcart P, Dickinson L, Efstathiou E, Emberton M, Fitzpatrick JM, Heidenreich A, Hughes S, Joniau S (2014). Management of prostate cancer in older patients: updated recommendations of a working group of the International Society of Geriatric Oncology. Lancet Oncol.

[5] Kirby M, Hirst C, Crawford ED (2011). Characterising the castration-resistant prostate cancer population: a systematic review. Int J Clin Pract.

[6] Taylor BS, Schultz N, Hieronymus H, Gopalan A, Xiao Y, Carver BS, Arora VK, Kaushik P, Cerami E, Reva B, Antipin Y, Mitsiades N, Landers T (2010). Integrative genomic profiling of human prostate cancer. Cancer Cell.

[7] Robinson D, Van Allen EM, Wu YM, Schultz N, Lonigro RJ, Mosquera JM, Montgomery B, Taplin ME, Pritchard CC, Attard G, Beltran H, Abida W, Bradley RK (2015). Integrative clinical genomics of advanced prostate cancer. Cell.

[8] Yu J, Yu J, Mani RS, Cao Q, Brenner CJ, Cao X, Wang X, Wu L, Li J, Hu M, Gong Y, Cheng H, Laxman B (2010). An integrated network of androgen receptor, polycomb, and TMPRSS2-ERG gene fusions in prostate cancer progression. Cancer Cell.

[9] Grasso CS, Wu YM, Robinson DR, Cao X, Dhanasekaran SM, Khan AP, Quist MJ, Jing X, Lonigro RJ, Brenner JC, Asangani IA, Ateeq B, Chun SY (2012). The mutational landscape of lethal castration-resistant prostate cancer. Nature.

[10] Park ES, Kim SJ, Kim SW, Yoon SL, Leem SH, Kim SB, Kim SM, Park YY, Cheong JH, Woo HG, Mills GB, Fidler IJ, Lee JS (2011). Cross-species hybridization of microarrays for studying tumor transcriptome of brain metastasis. Proc Natl Acad Sci U S A.

[11] Suraneni MV, Moore JR, Zhang D, Badeaux M, Macaluso MD, DiGiovanni J, Kusewitt D, Tang DG (2014). Tumor-suppressive functions of 15-Lipoxygenase-2 and RB1CC1 in prostate cancer. Cell Cycle.

[12] Sorensen KD, Abildgaard MO, Haldrup C, Ulhoi BP, Kristensen H, Strand S, Parker C, Hoyer S, Borre M, Orntoft TF (2013). Prognostic significance of aberrantly silenced ANPEP expression in prostate cancer. Br J Cancer.

[13] Larkin SE, Holmes S, Cree IA, Walker T, Basketter V, Bickers B, Harris S, Garbis SD, Townsend PA, Aukim-Hastie C (2012). Identification of markers of prostate cancer progression using candidate gene expression. Br J Cancer.

[14] Corcoran C, Rani S, O’Driscoll L (2014). miR-34a is an intracellular and exosomal predictive biomarker for response to docetaxel with clinical relevance to prostate cancer progression. Prostate.

[15] De Petrocellis L, Arroyo FJ, Orlando P, Schiano Moriello A, Vitale RM, Amodeo P, Sanchez A, Roncero C, Bianchini G, Martin MA, Lopez-Alvarado P, Menendez JC (2016). Tetrahydroisoquinoline-derived urea and 2,5-diketopiperazine derivatives as selective antagonists of the transient receptor potential melastatin 8 (TRPM8) channel receptor and antiprostate cancer agents. J Med Chem.

[16] Uysal-Onganer P, Kawano Y, Caro M, Walker MM, Diez S, Darrington RS, Waxman J, Kypta RM (2010). Wnt-11 promotes neuroendocrine-like differentiation, survival and migration of prostate cancer cells. Mol Cancer.

[17] Lerch MM, Halangk W (2006). Human pancreatitis and the role of cathepsin. B. Gut.

[18] Wang GP, Xu CS (2010). Pancreatic secretory trypsin inhibitor: more than a trypsin inhibitor. World J Gastrointest Pathophysiol.

[19] Marshall A, Lukk M, Kutter C, Davies S, Alexander G, Odom DT (2013). Global gene expression profiling reveals SPINK1 as a potential hepatocellular carcinoma marker. PLoS One.

[20] Ducreux M, Cuhna AS, Caramella C, Hollebecque A, Burtin P, Goere D, Seufferlein T, Haustermans K, Van Laethem JL, Conroy T, Arnold D, Committee EG (2015). Cancer of the pancreas: ESMO Clinical Practice Guidelines for diagnosis, treatment and follow-up. Ann Oncol.

[21] Twine NC, Stover JA, Marshall B, Dukart G, Hidalgo M, Stadler W, Logan T, Dutcher J, Hudes G, Dorner AJ, Slonim DK, Trepicchio WL, Burczynski ME (2003). Disease-associated expression profiles in peripheral blood mononuclear cells from patients with advanced renal cell carcinoma. Cancer Res.

[22] Flavin R, Pettersson A, Hendrickson WK, Fiorentino M, Finn S, Kunz L, Judson GL, Lis R, Bailey D, Fiore C, Nuttall E, Martin NE, Stack E (2014). SPINK1 protein expression and prostate cancer progression. Clin Cancer Res.

[23] Tomlins SA, Rhodes DR, Yu J, Varambally S, Mehra R, Perner S, Demichelis F, Helgeson BE, Laxman B, Morris DS, Cao Q, Cao X, Andren O (2008). The role of SPINK1 in ETS rearrangement-negative prostate cancers. Cancer Cell.

[24] Welsh M, Moffat L, McNeilly A, Brownstein D, Saunders PT, Sharpe RM, Smith LB (2011). Smooth muscle cell-specific knockout of androgen receptor: a new model for prostatic disease. Endocrinology.

[25] Lin C, Yin Y, Bell SM, Veith GM, Chen H, Huh SH, Ornitz DM, Ma L (2013). Delineating a conserved genetic cassette promoting outgrowth of body appendages. PLoS Genet.

[26] Kaushik AK, Vareed SK, Basu S, Putluri V, Putluri N, Panzitt K, Brennan CA, Chinnaiyan AM, Vergara IA, Erho N, Weigel NL, Mitsiades N, Shojaie A (2014). Metabolomic profiling identifies biochemical pathways associated with castration-resistant prostate cancer. J Proteome Res.

[27] Belanger A, Pelletier G, Labrie F, Barbier O, Chouinard S (2003). Inactivation of androgens by UDP-glucuronosyltransferase enzymes in humans. Trends Endocrinol Metab.

[28] Vainio P, Wolf M, Edgren H, He T, Kohonen P, Mpindi JP, Smit F, Verhaegh G, Schalken J, Perala M, Iljin K, Kallioniemi O (2012). Integrative genomic, transcriptomic, and RNAi analysis indicates a potential oncogenic role for FAM110B in castration-resistant prostate cancer. Prostate.

[29] Cai C, Wang H, He HH, Chen S, He L, Ma F, Mucci L, Wang Q, Fiore C, Sowalsky AG, Loda M, Liu XS, Brown M (2013). ERG induces androgen receptor-mediated regulation of SOX9 in prostate cancer. J Clin Invest.

[30] Olmos D, Brewer D, Clark J, Danila DC, Parker C, Attard G, Fleisher M, Reid AH, Castro E, Sandhu SK, Barwell L, Oommen NB, Carreira S (2012). Prognostic value of blood mRNA expression signatures in castration-resistant prostate cancer: a prospective, two-stage study. Lancet Oncol.

[31] Ross-Adams H, Lamb AD, Dunning MJ, Halim S, Lindberg J, Massie CM, Egevad LA, Russell R, Ramos-Montoya A, Vowler SL, Sharma NL, Kay J, Whitaker H (2015). Integration of copy number and transcriptomics provides risk stratification in prostate cancer: a discovery and validation cohort study. EBioMedicine.

[32] Robinson MD, McCarthy DJ, Smyth GK (2010). edgeR: a Bioconductor package for differential expression analysis of digital gene expression data. Bioinformatics.

[33] Venables WN, Ripley BD (2002). Modern applied statistics with S.

