# Supplementary material for: Transcriptomic features of primary prostate cancer and their prognostic relevance to castration-resistant prostate cancer
Source: Oncotarget. 2017 Nov 6;8(70):114845–55. doi: 10.18632/oncotarget.22296 (PMC5777737; doi:10.18632/oncotarget.22296)
Supplement: Supplementary file 1 [file oncotarget-08-114845-s001.pdf]

Transcriptomic features of primary prostate cancer and their prognostic relevance to castration-resistant prostate cancer

SUPPLEMENTARY MATERIALS

RNA extraction and RNA-Seq experiments

Total RNA was isolated by using the RNeasy Mini Kit (Qiagen, CA) according to the manufacturer's protocol. The quality and integrity of the RNA were confirmed by agarose gel electrophoresis and ethidium bromide staining followed by visual examination under ultraviolet light. The sequencing library was prepared by using the TruSeq RNA Sample Preparation kit V2 (Illumina, CA) according to the manufacturer's instructions. In brief, mRNA was purified from total RNA by using poly-T oligo-attached magnetic beads. The mRNA was then fragmented and converted into cDNA. Adapters were ligated to the cDNA and the fragments were amplified by PCR. Sequencing was performed on paired-end reads (2×100 bp) by using a Hiseq-2000 (Illumina).

RNA-Seq data processing

Reference genome sequence data from *Homo sapiens* were obtained from the University of California Santa Cruz Genome Browser Gateway (assembly ID: hg19). The reference genome index was built by using the Bowtie2-build component of Bowtie2 (ver. 2.0) and SAMtools (ver. 0.1.18). Tophat2 was applied to tissue samples for mapping reads to the reference genome (ver. 2.0). The statistics of the mapping activity of Tophat2 are shown in Supplementary Table 2. The dataset generated by RNA-Seq is available in the National Center for Biotechnology Information (NCBI) Gene Expression Omnibus (GEO) public database under the data series accession number GSE80609.

Public gene-expression datasets used for validation

Analysis of the NGS cohort and the four advanced prostate cancer (PC):casteration-resistant PC (CRPC) NGS sample pairs showed that 12 genes were differentially expressed between advanced PC (AdvPC) and CRPC. To validate these findings, five gene-expression datasets based on PC samples at various disease stages were selected from the National Center for Biotechnology (NCBI) Gene Expression Omnibus (GEO). The five datasets used were GSE28403, GSE32269, GSE35988, GSE37199, and GSE70768. GSE28403 contained the gene-expression data of four cases of AdvPC and nine cases of CRPC. All AdvPC and two CRPC samples were collected from distant metastatic sites; the remaining seven CRPC samples were acquired from the primary site on the prostate [1]. GSE32269 contained the gene-expression data of 22 localized PCs (hormone-sensitive) and 29 prostate-to-bone metastatic CRPCs [2]. GSE35988 consisted of 59 localized PCs and 35 metastatic CRPCs whose gene expression data were generated by two custom microarray platforms [3]. The gene-expression dataset in GSE37199 was generated from 107 PC samples obtained from 92 PC patients (31 good prognosis PCs and 63 advanced CRPCs) and included nine biological and four technical replicates. Thus, in total, GSE37199 contained 39 good prognosis PCs and 68 CRPCs [4]. Lastly, GSE70768 contained gene-expression data from 13 CRPCs and 113 PCs from the prostate that were collected by robotic radical prostatectomy [5]. The GSE28403, GSE32269, and GSE37199 gene-expression datasets were generated by using the Affymetrix Human Genome U133A and U133 Plus 2.0 arrays. The GSE35988 dataset was generated by using two customized Agilent platforms (Agilent Whole Human Genome Microarray G4112F and G4112A). The GSE70768 dataset was created by using an Illumina HumanHT-12 V4.0 expression bead chip. It should be noted that even after exploring various PC datasets in the public database, only a few were perfectly compatible with our unique RNA-Seq dataset.

Real-time PCR for validation of candidate genes

mRNA expression was measured by real-time polymerase chain reaction (PCR) by using a Rotor Gene 6000 instrument (Corbett Research, Mortlake, Australia). Real-time PCR was performed in microreaction tubes (Corbett Research) with SsoFast EvaGreen Supermix (Bio-Rad Laboratories, Hercules, CA, USA). The primers used to amplify mRNA for *AR*, *SPINK1*, *SERPINB11*, *SP8*, *CBNL*, *CEACAM20*, *CEACAM22P*, and *GAPDH* from tissues were as follows: *AR* sense, 5'-TCC ATC TTG TCG TCT TCG GA-3', and *AR* antisense, 5'-TGT GAC ACT GTC AGC TTC TG-3'; *SPINK1* sense, 5'-GAA CTT AAT GGA TGC ACC AAG-3', and *SPINK1* antisense, 5'-TCA GCA AGG CCC AGA TTT TT-3'; *SERPINB11*

sense, 5'-TTG TAA AGG AGC CGC AGA TG-3', and *SERPINB11* antisense, 5'-GGT TGA AGA GAT CTG TCA CC-3'; *SP8* sense, 5'-ATG CTT GCC GCT ACC TGT AA-3', and *SP8* antisense, 5'-AGA CTG GAA CCC ACT ACG TT-3'; *CBLN* sense, 5'-TTG TAA AGG AGC CGC AGA TG-3', and *CBLN* antisense, 5'-CAT TAA ACT GAC CTG GAT GG-3'; *CEACAM20* sense, 5'-AG CCT CGC TTT GTA CCG TA-3', and *CEACAM20* antisense, 5'-AGA AGG GCA TCT CGA GCT TC-3'; *CEACAM22P* sense, 5'-GAC CAG TAA TCT CCA GAG AT-3', and *CEACAM22P* antisense, AGC AGA CCA GAG TGA GGT TG; *GAPDH* 5'-AGC AGA CCA GAG TGA GGT TG-3'; and sense, 5'-CAT GTT CGT CAT GGG TGT GA-3' and antisense, 5'-ATG GCA TGG ACT GTG GTC AT-3'. The real-time PCR conditions used to amplify *AR*, *SPINK1*, *SP8*, *CEACAM22P*, and *GAPDH* from PC tissue specimens were as follows: 1 cycle for 2 seconds at 96ºC, and then 45 cycles of 2 seconds at 96ºC (denaturation), 20 seconds at 60ºC (annealing), and 20 seconds at 72ºC (extension). For *SERPINB11*, *CBLN*, and *CEACAM20*, the conditions were 1 cycle for 2 seconds at 96ºC, followed by 45 cycles of 2 seconds at 96ºC for denaturation, 20 seconds at 54ºC for annealing, and 20 seconds at 72ºC for extension. The melting program was performed at 65ºC–95ºC with a heating rate of 1ºC per 45 seconds. Expression of these genes was normalized to that of *GAPDH*, which was analyzed in parallel. All samples were run as triplicates.

SUPPLEMENTARY RESULTS

Biological insights into the signature gene profile of progression to CRPC

To identify signaling pathways that participate in the progression of AdvPC to CRPC, function enrichment test and gene-to-gene network analyses were performed with the 90 genes that were differentially expressed between AdvPC and CRPC in the NGS analysis (Figure 1). For this, the IPA tool was used. As expected, when we searched for enriched functions, there was significant enrichment of genes involved in cancer, cellular growth and proliferation, the cell cycle, and cell death and survival. We also found high enrichment of genes involved in immunological and inflammatory disease (Supplementary Figure 7). Interestingly, the vast majority of enriched functions contained *AR* or *SPINK1*, which are the two oncogenic molecules that were found to be up-regulated in CRPCs relative to in AdvPC when we compared the paired and unpaired AdvPC:CRPC samples in our NGS cohort. This indicates that *AR* and *SPINK1* play a crucial role in the progression of AdvPC to CRPC.

An exploration of gene-to-gene networks revealed a functional connectivity between *AR* and *SPINK1* in a network in which *SPINK1* is a downstream effector of *AR* [6] (Figure 4). *SPINK1* plays a number of roles in the cell, including in abnormal morphology and proliferation [7, 8]. It also regulates many matrix metalloproteinase family genes, including *MMP13*, which participates in the identified network (Figure 4). Of the 90 candidate genes identified by the NGS analysis, we also found putative activation of the oncogenic transcription regulator *HNF1A* (Figure 4). *HNF1A* formed the primary hub of the gene network we identified because it regulates many downstream effectors, including UDP glucuronosyltransferase family members (*i.e.*, *UGT1A1*, *UGT1A3*, and *UGT2B15*), *VIL1*, *AKR1C1*/*AKR1C2*, and *ANPEP*. *HNF1A* is well-known to participate in several molecular functions, including cancer, cell proliferation, cell or tumor morphology, and gastrointestinal disease.

To confirm the activities of these *AR*- and *HNF1A*-connected candidate genes, we examined their expression levels in CRPC relative to those in controls by using five publicly available datasets that are based on independent patient cohorts, namely, GSE28403, GSE32269, GSE35988, GSE37199, and GSE70768. The controls in these comparisons were AdvPC (GSE28403), localized PC (GSE32269 and GSE35988), PC with a good prognosis (GSE37199), and primary-site PC (GSE70768). In these analyses, we focused on the network molecules that our NGS analysis showed were up-regulated in CRPC relative to AdvPC (*i.e., AR*, *SPINK1*, *S100A8*, *HNF1A*, *VIL1*, and *MMP13*). In total, 54 comparisons were made in these five datasets. All six genes were generally up-regulated in CRPC compared to in the control. Many of these up-regulations were significant. In only three cases, down-regulation was observed (once for *AR* and twice for *HNF1*) (Supplementary Figures 8–12). These data support the notion that gene networks that are mediated by *AR* or *HNF1A* participate in the development of CRPC.

REFERENCES

1. Vainio P, Wolf M, Edgren H, He T, Kohonen P, Mpindi JP, Smit F, Verhaegh G, Schalken J, Perala M, Iljin K, Kallioniemi O. Integrative genomic, transcriptomic, and RNAi analysis indicates a potential oncogenic role for FAM110B in castration-resistant prostate cancer. Prostate. 2012; 72:789-802. https://doi.org/10.1002/pros.21487.
2. Cai C, Wang H, He HH, Chen S, He L, Ma F, Mucci L, Wang Q, Fiore C, Sowalsky AG, Loda M, Liu XS, Brown M, et al. ERG induces androgen receptor-mediated regulation of SOX9 in prostate cancer. J Clin Invest. 2013; 123:1109-22. https://doi.org/10.1172/JCI66666.
3. Grasso CS, Wu YM, Robinson DR, Cao X, Dhanasekaran SM, Khan AP, Quist MJ, Jing X, Lonigro RJ, Brenner JC, Asangani IA, Ateeq B, Chun SY, et al. The mutational landscape of lethal castration-resistant prostate cancer. Nature. 2012; 487:239-43. https://doi.org/10.1038/nature11125.
4. Olmos D, Brewer D, Clark J, Danila DC, Parker C, Attard G, Fleisher M, Reid AH, Castro E, Sandhu SK, Barwell L, Oommen NB, Carreira S, et al. Prognostic value of blood mRNA expression signatures in castration-resistant prostate cancer: a prospective, two-stage study. Lancet Oncol. 2012; 13:1114-24. https://doi.org/10.1016/S1470-2045(12)70372-8.
5. Ross-Adams H, Lamb AD, Dunning MJ, Halim S, Lindberg J, Massie CM, Egevad LA, Russell R, Ramos-Montoya A, Vowler SL, Sharma NL, Kay J, Whitaker H, et al. Integration of copy number and transcriptomics provides risk stratification in prostate cancer: a discovery and validation cohort study. EBioMedicine. 2015; 2:1133-44. https://doi.org/10.1016/j.ebiom.2015.07.017.
6. Welsh M, Moffat L, McNeilly A, Brownstein D, Saunders PT, Sharpe RM, Smith LB. Smooth muscle cell-specific knockout of androgen receptor: a new model for prostatic disease. Endocrinology. 2011; 152:3541-51. https://doi.org/10.1210/en.2011-0282.
7. Ohmuraya M, Hirota M, Araki M, Mizushima N, Matsui M, Mizumoto T, Haruna K, Kume S, Takeya M, Ogawa M, Araki K, Yamamura K. Autophagic cell death of pancreatic acinar cells in serine protease inhibitor Kazal type 3-deficient mice. Gastroenterology. 2005; 129:696-705. https://doi.org/10.1016/j.gastro.2005.05.057.
8. Wang GP, Xu CS. Pancreatic secretory trypsin inhibitor: more than a trypsin inhibitor. World J Gastrointest Pathophysiol. 2010; 1:85-90. https://doi.org/10.4291/wjgp.v1.i2.85.

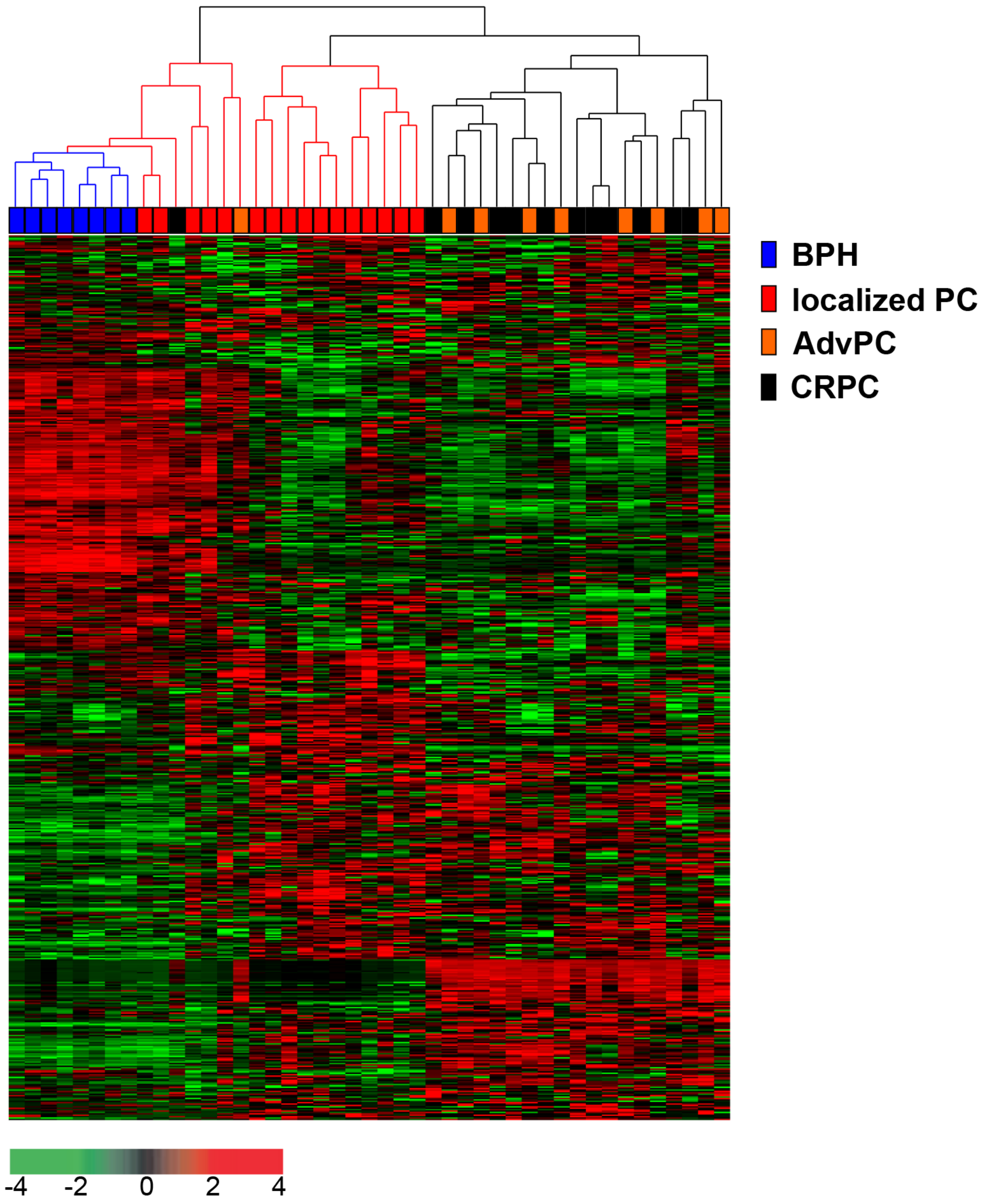

Supplementary Figure 1: Gene expression pattern of prostate cancer in the NGS cohort (n=45). Gene expression patterns were obtained by performing hierarchical cluster analysis. Benign prostate hyperplasia (BPH), localized primary prostate cancer (PC), advanced PC (AdvPC), and castration-resistant prostate cancers (CRPC) were applied to cluster analysis. Genes with an expression ratio that has at least a two-fold difference relative to the median gene expression level across all tissues in at least 10 tissues were selected for hierarchical analysis (1,274 gene features). The red and green colors reflect high and low expression levels, respectively. NGS, next generation sequencing.

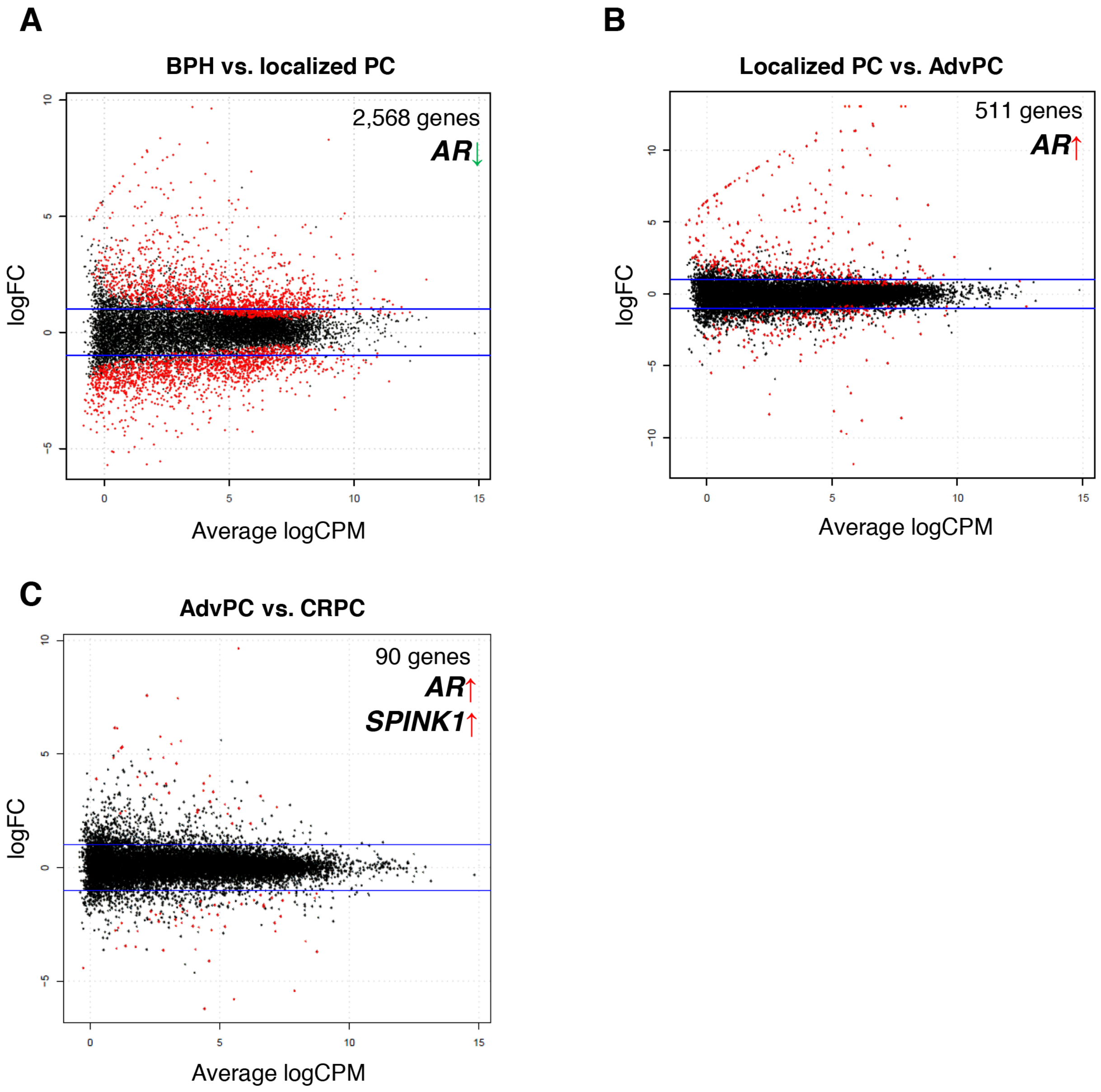

Supplementary Figure 2: Bland-Altman plots of differentially expressed genes between two prostate tissue types. Genes were scattered based on their average expression values and fold ratio **(A)** between benign prostate hyperplasia (BPH) and localized primary prostate cancer (PC), **(B)** between localized PC and advanced PC (AdvPC), and **(C)** between AdvPC and castration-resistant prostate cancer (CRPC), respectively. Red points indicate statistically significant genes tested by the GLM likelihood ratio test in EdgeRsoftware. Cut-off criteria of a *P*-value of less than 0.001. logFC, log2-transformed fold change. logCPM, log2-transformed counts per million mapped reads.

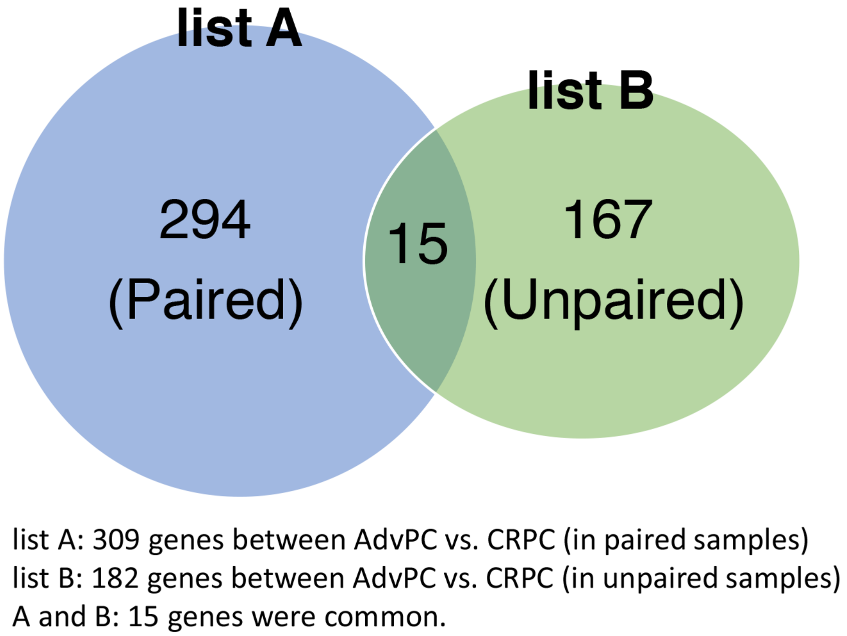

Supplementary Figure 3: Comparative analysis of significant gene lists in matched sample pairs and in unpaired remained samples. Venn diagram of genes selected by the generalized linear model likelihood ratio test using EdgeRsoftware was applied. Genes in the blue circle (gene list **A**) represent those differentially expressed between AdvPC and CRPC in paired samples. Genes in the green circle (gene list **B**) represent those differentially expressed between AdvPC and CRPC in unpaired samples. Cut-off criteria of a *P*-value of less than 0.001 and a 2-fold or greater relative difference were applied to select genes whose expression were significantly different between the two groups. AdvPC, advanced prostate cancer. CRPC, castration-resistant prostate cancer.

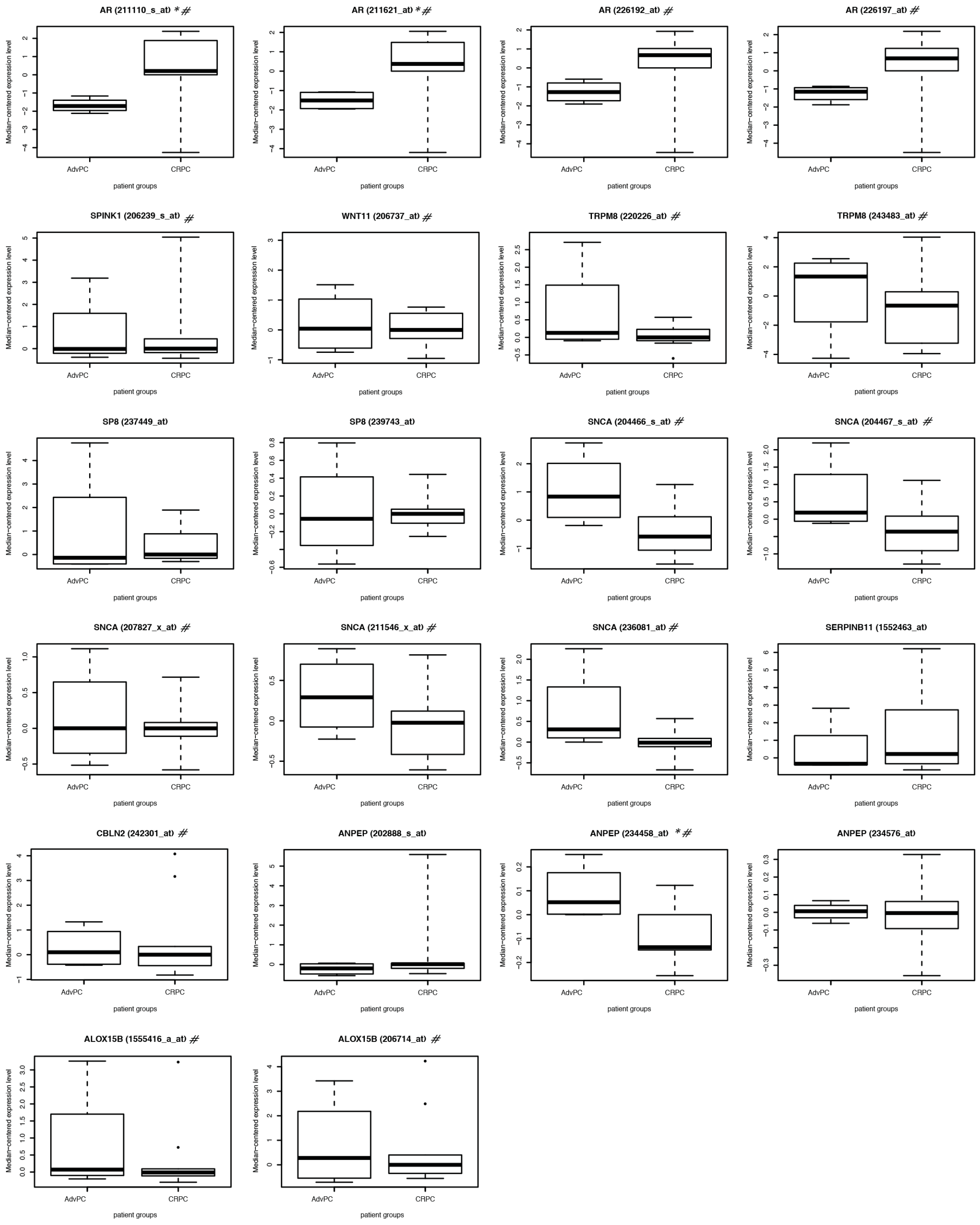

Supplementary Figure 4: Expression levels of prostate cancers in a public data set (GSE28403). Two group box plots comparing expression levels of the genes associated with CRPC development between AdvPC and CRPC groups. Accession number in parenthesis means probe id of microarray platform. # indicates positively correlated with average gene expression changes in the NGS cohort. AdvPC, advanced prostate cancer. CRPC, castration-resistant prostate cancer. NGS, next generation sequencing. *, $P < 0.05$. P-value was obtained by two-sample t-test.

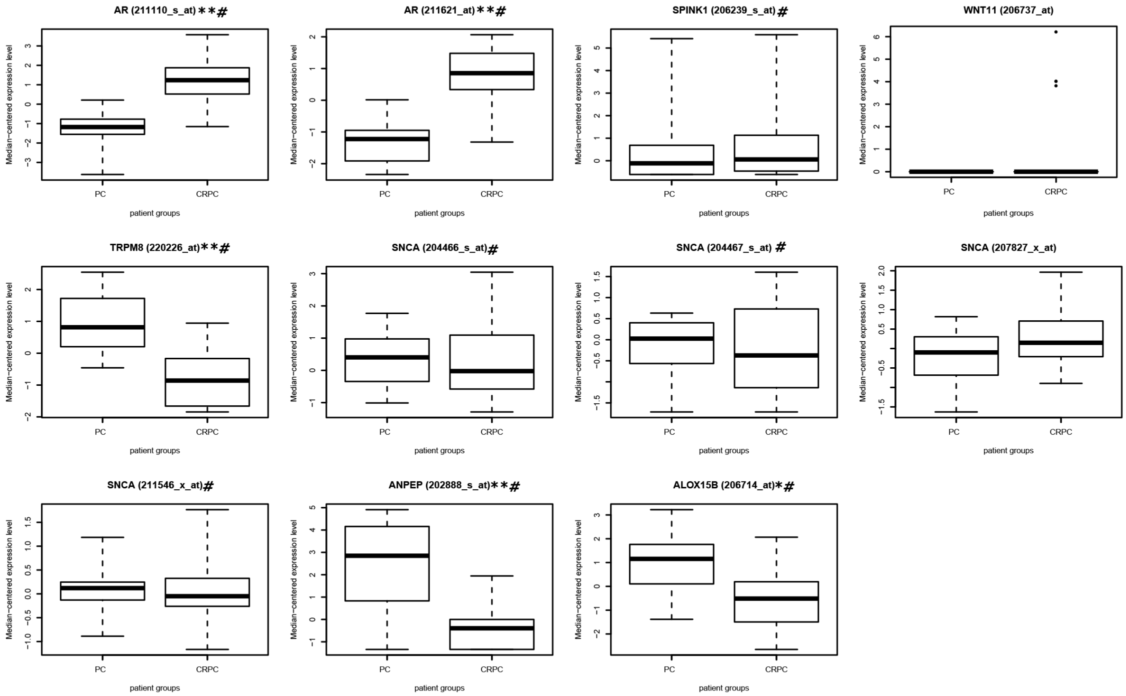

Supplementary Figure 5: Expression levels of prostate cancers in a public data set (GSE32269). Two group box plots comparing expression levels of the genes associated with CRPC development between localized PC and CRPC groups. Accession number in parenthesis means probe id of microarray platform. # indicates positively correlated with average gene expression changes in the NGS cohort. PC, prostate cancer. CRPC, castration-resitant prostate cancer. NGS, next generation sequencing. *, $P < 0.05$. **, $P < 0.001$. P-value was obtained by two-sample t-test.

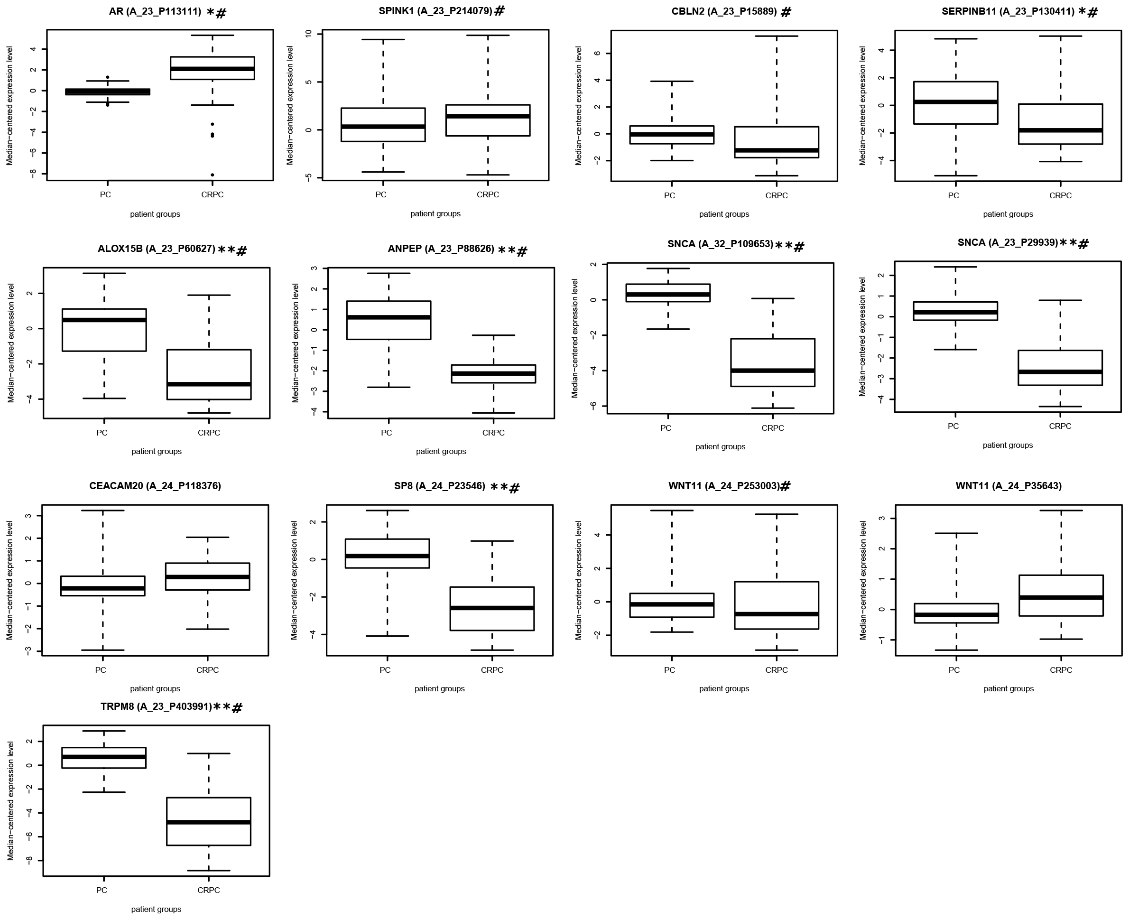

Supplementary Figure 6: Expression levels of prostate cancers in a public data set (GSE35988). Two group box plots comparing expression levels of the genes associated with CRPC development between localized PC and metastatic CRPC groups. Accession number in parenthesis means probe id of microarray platform. # indicates positively correlated with average gene expression changes in the NGS cohort. PC, prostate cancer. CRPC, castration-refractory prostate cancer. NGS, next generation sequencing. *, $P < 0.05$. **, $P < 0.001$. P-value was obtained by two-sample t-test.

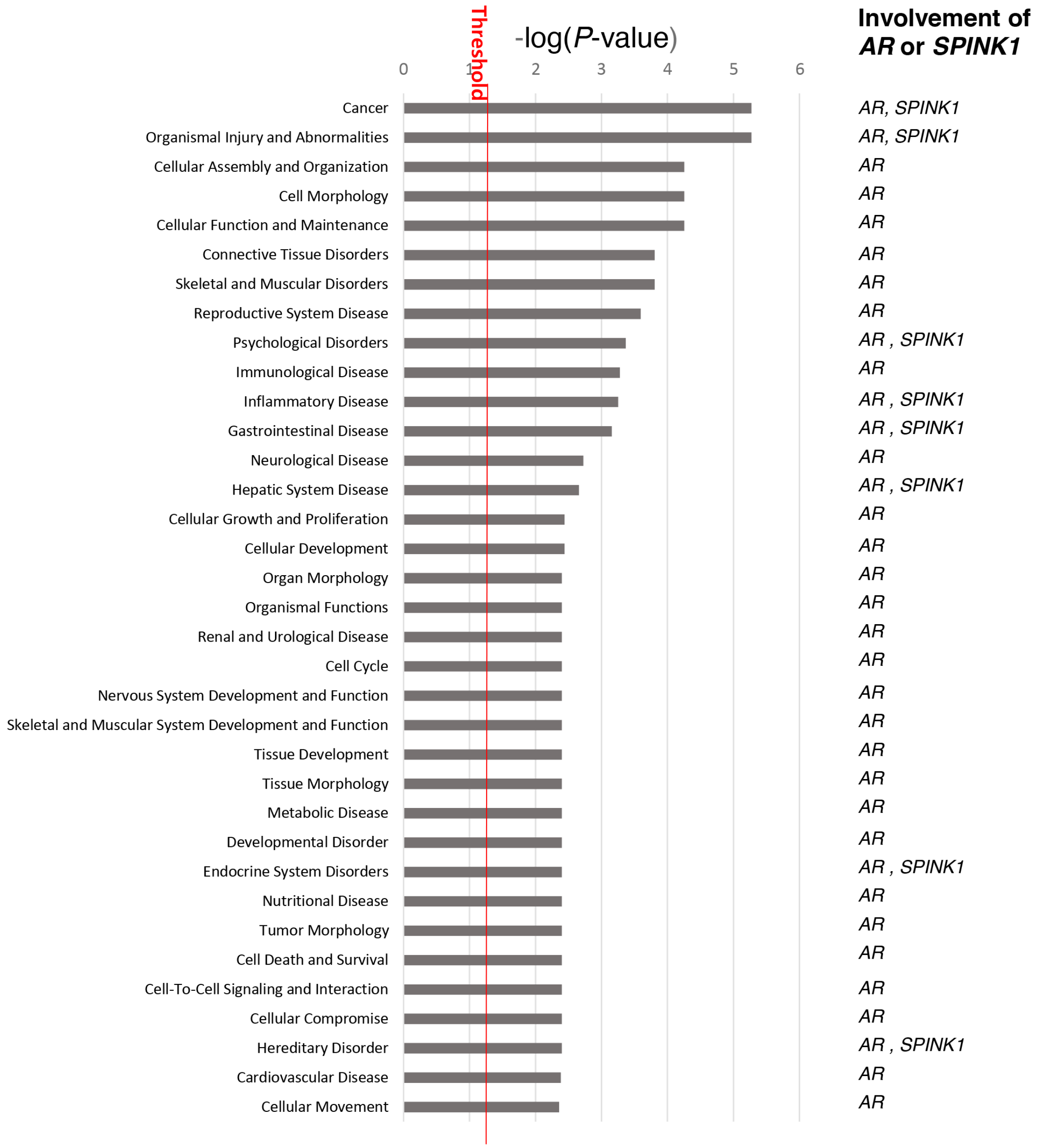

Supplementary Figure 7: Gene set enrichments analysis of CRPC progression associated genes. Enriched gene set terms were determined using Ingenuity Pathway Analysis software. The threshold of significance was $P < 0.05$.

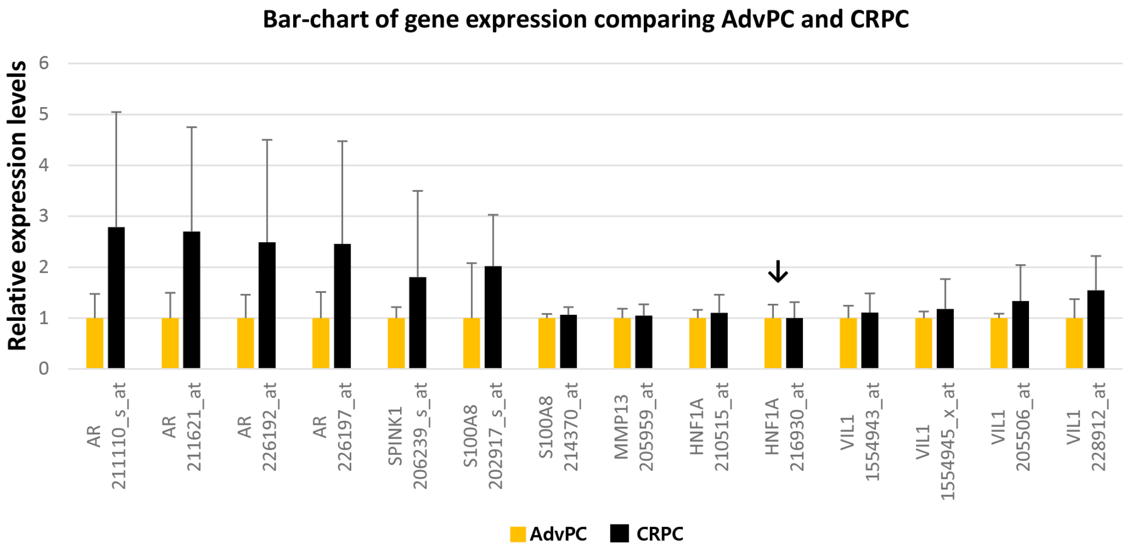

Supplementary Figure 8: Expression levels of prostate cancers in a public data set (GSE28403). Bar-plots comparing expression levels of the genes associated with CRPC development between AdvPC and CRPC groups. "↓" indicates down-regulated in the CRPC subgroup. AdvPC, advanced prostate cancer. CRPC, castration-resistance prostate cancer.

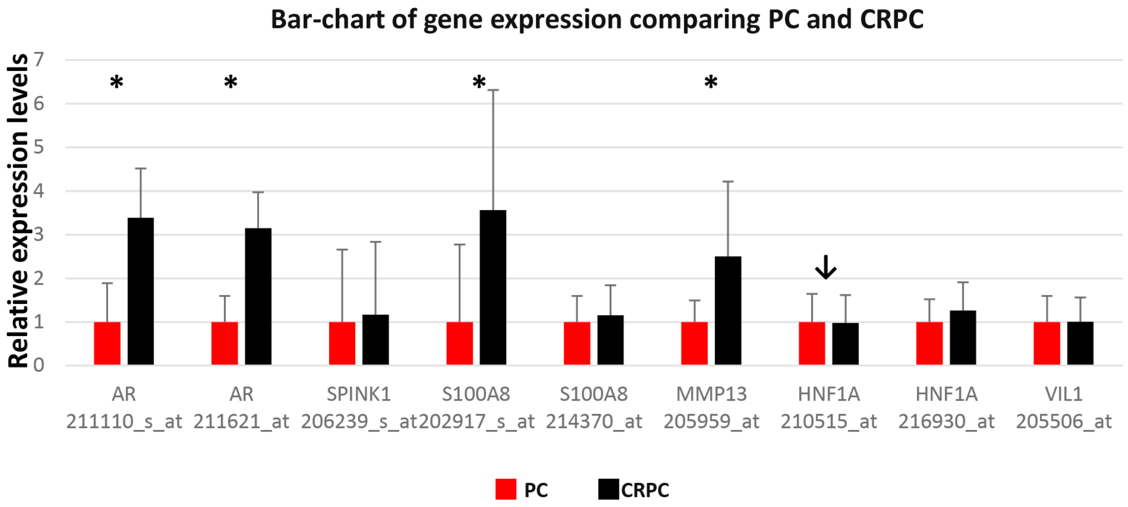

Supplementary Figure 9: Expression levels of prostate cancers in a public data set (GSE32269). Bar-plots comparing expression levels of the genes associated with CRPC development between localized PC and CRPC groups. "↓" indicates down-regulated in the CRPC subgroup. PC, prostate cancer. CRPC, castration-resistant prostate cancer. *, $P < 0.05$. P-value was obtained by two-sample t-test.

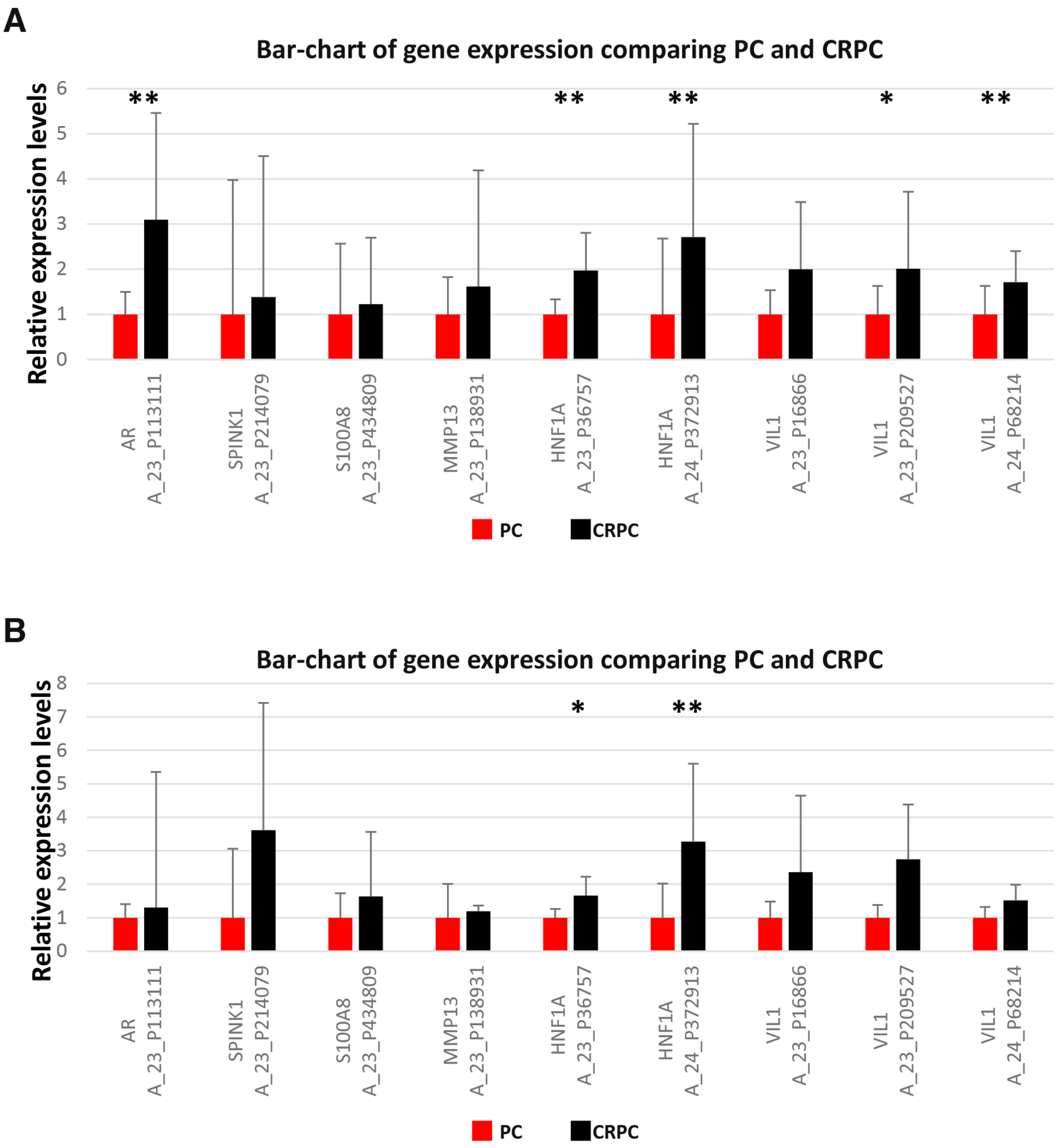

Supplementary Figure 10: Expression levels of prostate cancers in a public data set (GSE35988). Bar-plots comparing expression levels of the genes associated with CRPC development between localized PC and CRPC groups in **(A)** GPL6480 and **(B)** GPL6848 platforms. PC, prostate cancer. CRPC, castration-refractory prostate cancer. *, $P< 0.05$. **, $P< 0.001$. *P*-value was obtained by two-sample t-test.

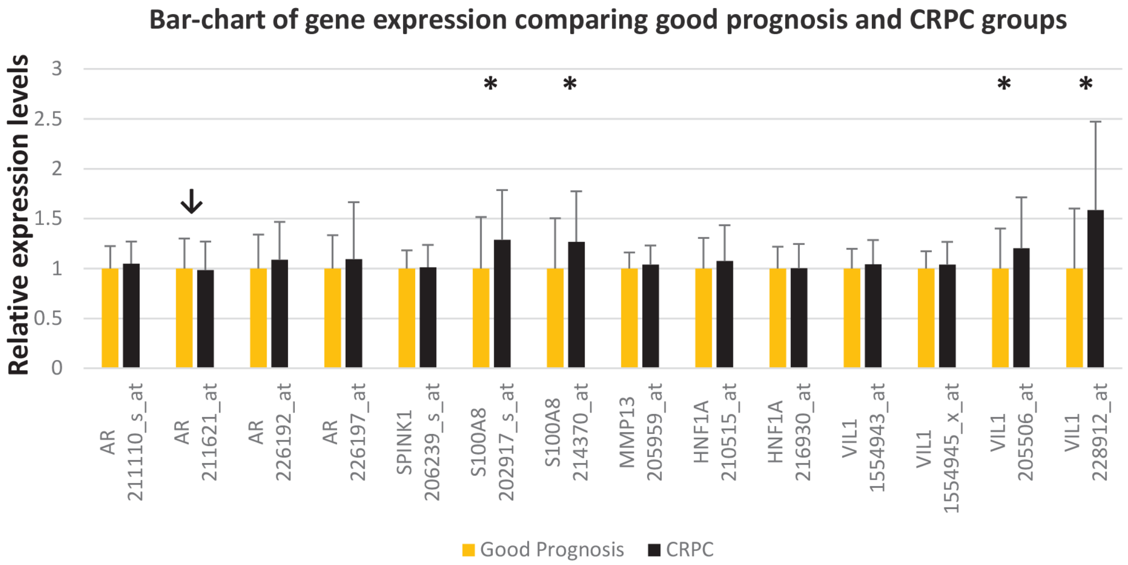

Supplementary Figure 11: Expression levels of prostate cancers in a public data set (GSE37199). Bar-plots comparing expression levels of the genes associated with CRPC development between good prognosis and CRPC patient groups in blood samples. "↓" indicates down-regulated in the CRPC subgroup. CRPC, castration-resistant prostate cancer. *, *P*< 0.05. *P*-value was obtained by two-sample t-test.

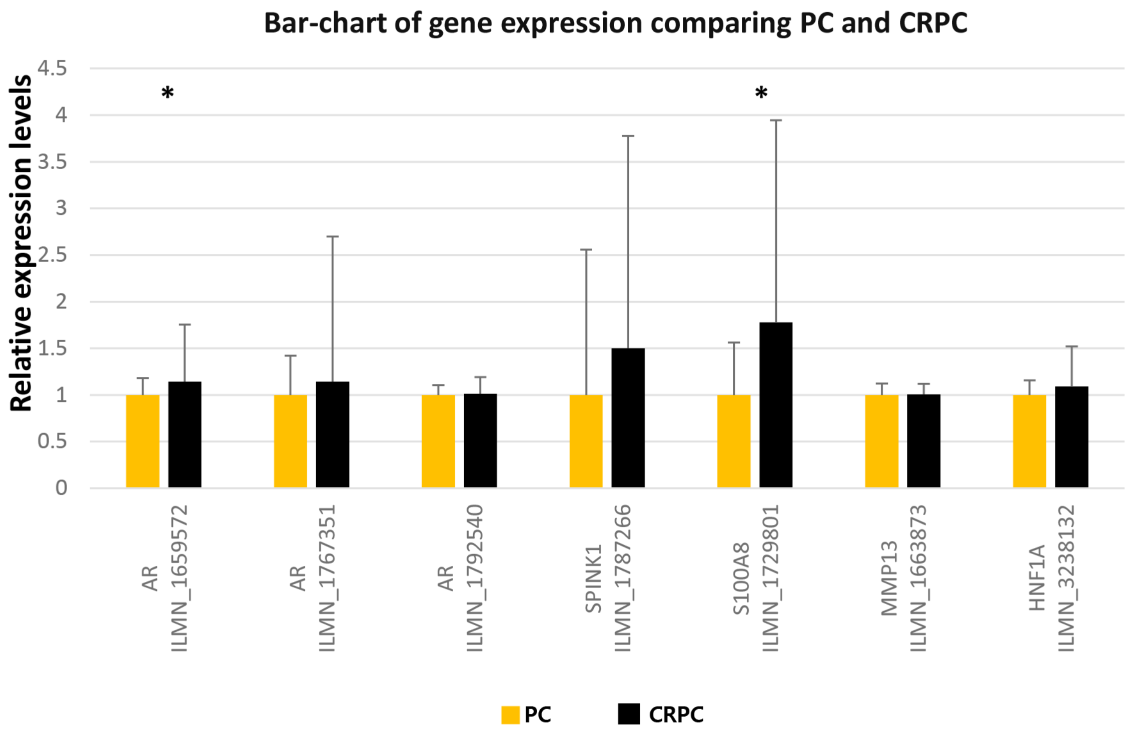

Supplementary Figure 12: Expression levels of prostate cancers in a public data set (GSE70768). Bar-plots comparing expression levels of the genes associated with CRPC development between PC and CRPC patient groups. PC, prostate cancer. CRPC, castration-resistant prostate cancer. *, $P < 0.05$. P-value was obtained by two-sample t-test.

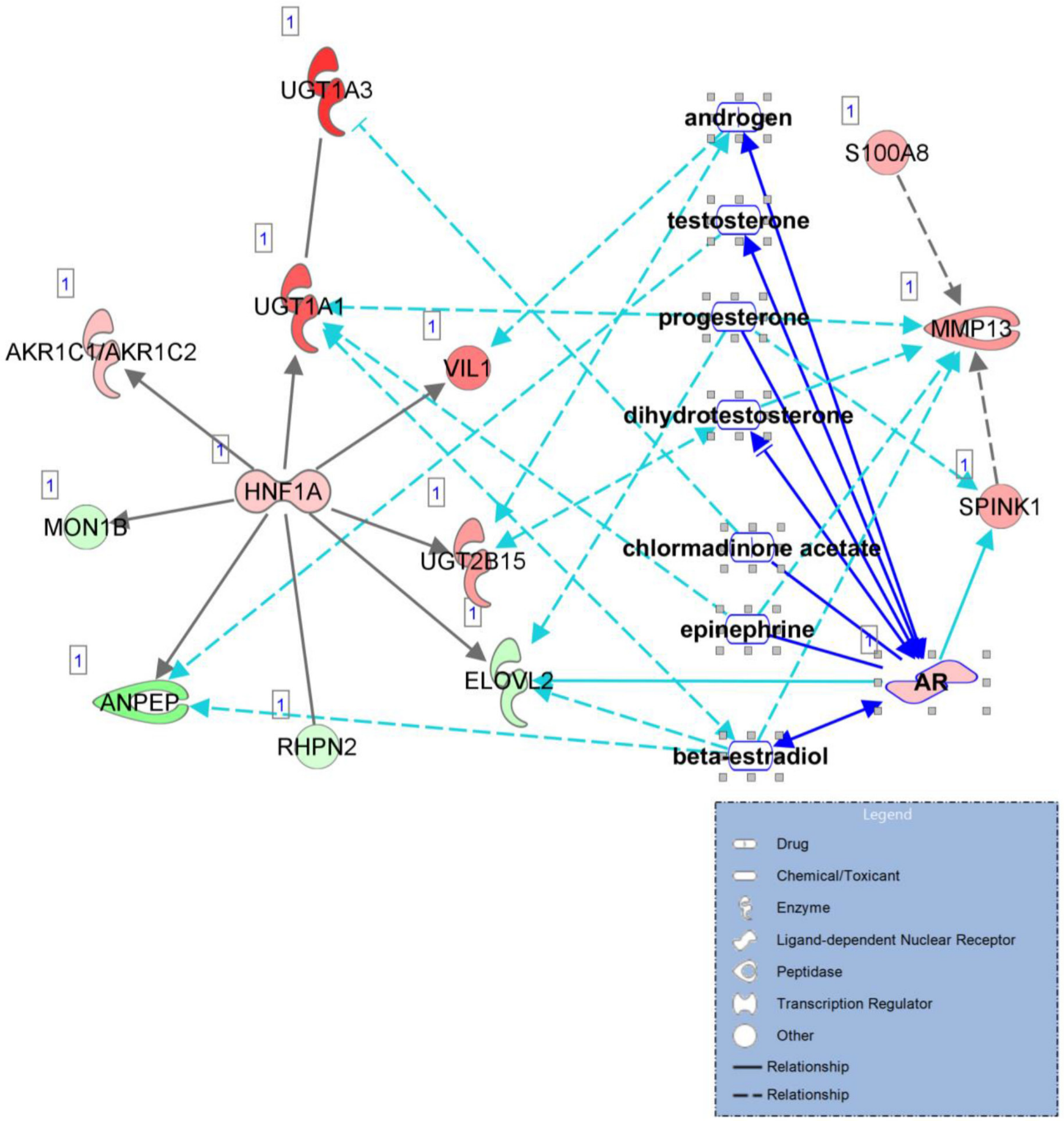

Supplementary Figure 13: Extended gene network analysis displaying interactions between the CRPC progression associated genes and hormones. Gene networks were explored using 90 genes that differentially expressed genes between AdvPC and the CRPC with *AR*-interactive hormones. Up-and down-regulated genes in the CRPC subgroup are indicated in red and green, respectively. The intensity of color is indicative of the degree of over-or under-expression. Each line and arrow represents functional and physical interactions between the genes and the direction of regulation reported in the literature. AdvPC, advanced prostate cancer. CRPC, castration-resistant prostate cancer.

Supplementary Table 1: Clinical course of the four patients in the NGS cohort who had an AdvPC biopsy followed by a CRPC biopsy

Case no.	1st Operation						2nd Operation				
	Age	Type of surgery	PSA	Gleason score	TNM stage	Time to CRPC (months)	Age	Type of surgery	PSA	Gleason score	TNM stage
1	69	RRP	90.8	7 (3+4)	T3N1M0	124.7	81	TUR-P	133.1	7 (4+3)	T4N1M1
2	74	TUR-P	379.0	7 (4+3)	T3N1M1	26.2	78	TUR-P	166.0	7 (4+3)	T3N1M1
3	78	TUR-P	200.0	9 (4+5)	T3N1M0	13.8	79	TUR-P	40.2	9 (5+4)	T3N1M0
4	69	TUR-P	89.0	9 (4+5)	T4N1M0	9.3	70	TUR-P	117.3	9 (4+5)	T4N1M0

Abbreviations: AdvPC, advanced prostate cancer; CRPC, castration-resistant prostate cancer; NGS, next-generation sequencing; PSA, prostate-specific antigen; RRP, radical retropubic prostatectomy; TUR-P, transurethral resection of the prostate.

Supplementary Table 2: Mapping rates to the *Homo sapiens* reference genome by Tophat2 in the NGS cohort

Patient ID	Tissue type	# mapped reads	# unmapped reads	Mapping ratios (%)
**H726**	BPH	15,994,941	7,276,439	68.7%
**H731**	BPH	15,378,262	6,553,472	70.1%
**H732**	BPH	25,899,812	12,409,448	67.6%
**H735**	BPH	21,519,964	9,689,804	69.0%
**H736**	BPH	37,972,019	17,081,725	69.0%
**H745**	BPH	46,916,461	23,849,229	66.3%
**H747**	BPH	20,914,425	9,428,889	68.9%
**H750**	BPH	17,710,692	8,733,686	67.0%
**RP097**	Localized PC	15,209,797	10,128,655	60.0%
**RP106**	Localized PC	17,086,017	11,582,275	59.6%
**RP166**	Localized PC	16,981,289	10,695,617	61.4%
**RP194**	Localized PC	20,157,993	13,394,333	60.1%
**RP280**	Localized PC	30,181,363	20,417,371	59.6%
**RP286**	Localized PC	16,995,206	11,226,446	60.2%
**RP342**	Localized PC	25,276,203	11,994,971	67.8%
**RP395**	Localized PC	23,834,391	11,826,713	66.8%
**RP432**	Localized PC	27,978,757	13,528,447	67.4%
**RP436**	Localized PC	31,739,247	14,924,579	68.0%
**RP437**	Localized CP	28,482,081	13,917,341	67.2%
**RP461**	Localized PC	39,460,373	19,368,623	67.1%
**RP463**	Localized PC	38,719,122	18,278,160	67.9%
**RP482**	Localized PC	28,591,951	14,087,891	67.0%
**RP496**	Localized PC	22,279,531	15,050,451	59.7%
**RP499**	Localized PC	15,906,502	10,827,342	59.5%
**RP264**	AdvPC	11,973,886	4,123,158	74.4%
**RP484**	AdvPC	10,250,695	3,678,987	73.6%
**RP553**	AdvPC	10,407,388	3,816,624	73.2%
**RP588**	AdvPC	9,873,038	3,529,706	73.7%
**RP621**	AdvPC	15,008,355	3,199,191	82.4%
**RP160**	AdvPC	18,827,736	4,581,680	80.4%
**RP516**	AdvPC	17,828,552	5,174,192	77.5%
**RP542**	AdvPC	18,819,675	5,557,045	77.2%
**RP62**	AdvPC	13,406,642	4,763,494	73.8%
**RP160+**	CRPC	18,743,673	4,813,405	79.6%
**RP516+**	CRPC	37,488,274	14,279,668	72.4%
**RP542+**	CRPC	9,889,280	3,503,332	73.8%
**RP62+**	CRPC	21,217,451	5,696,825	78.8%
**RP170**	CRPC	24,843,371	8,457,473	74.6%
**RP226**	CRPC	18,114,401	4,353,769	80.6%
**RP256**	CRPC	27,944,909	9,637,987	74.4%
**RP308**	CRPC	31,224,250	11,693,276	72.8%
**RP407**	CRPC	12,378,314	4,472,862	73.5%
**RP537**	CRPC	22,387,454	5,035,682	81.6%
**RP627**	CRPC	18,727,652	5,144,690	78.4%
**RP680**	CRPC	26,522,537	8,900,425	74.9%

Abbreviations: BPH, benign prostate hyperplasia; PC, prostate cancer; AdvPC, advanced PC; CRPC, castration-resistant PC.

Supplementary Table 3: Log2-transformed fold change values of genes that are differentially expressed between AdvPC and CRPC in the paired and unpaired samples

Genes	CRPC *vs*. AdvPC (paired samples)		CRPC *vs*. AdvPC (unpaired samples)	
	logFC	*P*-value	logFC	*P*-value
ALOX15B	-3.778	6.34×10^{-4}	-7.072	8.44×10^{-17}
ANPEP	-2.962	7.48×10^{-5}	-3.730	1.09×10^{-4}
AR	3.847	1.02×10^{-8}	1.826	5.08×10^{-5}
CBLN2	-3.271	1.42×10^{-4}	-6.687	4.70×10^{-5}
CEACAM20	-4.196	1.03×10^{-7}	-6.430	2.18×10^{-7}
CEACAM22P	-2.867	1.38×10^{-4}	-6.414	5.29×10^{-11}
KLK1	-3.447	9.00×10^{-6}	8.286	6.37×10^{-6}
PGC	-3.705	1.36×10^{-4}	4.067	4.41×10^{-6}
PPFIA2	3.414	2.34×10^{-6}	-3.188	1.72×10^{-4}
SERPINB11	-3.490	5.12×10^{-4}	-6.513	1.56×10^{-6}
SNCA	-2.528	9.06×10^{-5}	-3.057	2.98×10^{-5}
SP8	-5.151	4.56×10^{-6}	-5.363	1.63×10^{-5}
SPINK1	4.259	4.71×10^{-5}	4.355	1.61×10^{-5}
TRPM8	-2.852	8.73×10^{-6}	-6.156	1.79×10^{-10}
WNT11	-2.587	5.00×10^{-4}	-3.605	1.29×10^{-5}

P-values were obtained by the GLM likelihood ratio test provided by EdgeR package.
Abbreviations: AdvPC, advanced prostate cancer; CRPC, castration-resistant prostate cancer; logFC, log2-transformed fold change.
